# Person-related factors associated with work participation in employees with health problems: a systematic review

**DOI:** 10.1007/s00420-018-1308-5

**Published:** 2018-04-26

**Authors:** Mariska de Wit, Haije Wind, Carel T. J. Hulshof, Monique H. W. Frings-Dresen

**Affiliations:** Department Coronel Institute of Occupational Health, Academic Medical Center, University of Amsterdam, Amsterdam Public Health research institute, PO Box 22700, 1100 DE Amsterdam, The Netherlands

**Keywords:** Perceptions, Cognitions, Person-related factors, Work participation

## Abstract

**Purpose:**

The objective of this systematic review was to explore and provide systematically assessed information about the association between person-related factors and work participation of people with health problems. The research question was: what is the association between selected person-related factors and work participation of workers with health problems?

**Methods:**

A systematic review was carried out in PubMed and PsycINFO to search for original papers published between January 2007 and February 2017. The risk of bias of the studies included was assessed using quality assessment tools from the Joanna Briggs Institute. The quality of evidence was assessed using the GRADE framework for prognostic studies.

**Results:**

In total, 113 studies were included, all of which addressed the association between person-related factors and work participation. The factors positively associated with work participation were positive expectations regarding recovery or return to work, optimism, self-efficacy, motivation, feelings of control, and perceived health. The factors negatively associated with work participation were fear-avoidance beliefs, perceived work-relatedness of the health problem, and catastrophizing. Different coping strategies had a negative or a positive relationship with work participation.

**Conclusions:**

The results of this review provide more insight into the associations between different cognitions and perceptions and work participation. The results of this study suggest that person-related factors should be considered by occupational- and insurance physicians when they diagnose, evaluate or provide treatment to employees. Further research is required to determine how these physicians could obtain and apply such information and whether its application leads to a better quality of care.

**Electronic supplementary material:**

The online version of this article (10.1007/s00420-018-1308-5) contains supplementary material, which is available to authorized users.

## Introduction

Sickness absence has negative financial consequences and leads to a loss of enthusiasm and satisfaction with the work situation (Sieurin et al. 2009). In addition, long-term sick leave can lead to lower self-confidence, a depressed mood and feelings of isolation (Bryngelson 2009; Vingård et al. 2004). These negative consequences of sick leave constitute significant reasons why it is important to minimize the work absence of employees due to health problems.

In order to minimize work absence and improve work participation, it is essential to know which factors influence work retention and return to work (RTW) after sick leave. Research has revealed that sick leave is determined by many different factors (Dekkers-Sánchez et al. 2013; World Health Organization 2001). In addition to disease-related and environmental factors, person-related factors such as cognitions and perceptions of employees also play a role in work participation (Dekkers-Sánchez et al. 2013; Iles et al. 2008; Vooijs et al. 2015). Research by Dekkers-Sánchez et al. (2013) has revealed that physicians identify person-related factors as important factors for RTW. The cognitions and perceptions of an employee about his or her health problems or limitations, are factors in which clinicians could intervene to encourage work participation (Dekkers-Sánchez et al. 2013; Verbeek 2006).

As most research acknowledges the multifactorial nature of sick leave, many reviews have been conducted to gain better insight into the precise factors influencing the work participation of employees with health problems. However, most of these reviews are limited to specific diseases or disorders, or are limited to the outcome RTW rather than work participation in general (Blank et al. 2008; Clay et al. 2010; Van Velzen et al. 2009). In addition, as far as we know, there is no review which primarily focuses on the cognitions and perceptions of employees themselves that influence work participation. This is despite the fact that structuring the information about the influence of cognitions and perceptions could help to develop tailored interventions targeting these factors. Such interventions could in turn be used to support work participation of employees with health problems (Dekkers-Sánchez et al. 2013). Moreover, a clear overview of the association between person-related factors and work participation could assist occupational physicians and insurance physicians to prevent sick leave or decrease the duration of sick leave in these employees.

This systematic review was conducted to fill this gap in research and provide structured information about the association between person-related factors and work participation for employees with all kinds of diseases, disorders and injuries. For this review of the literature, we formulated the following research question using the patient, intervention, comparison, outcome (PICO) statement: in employees with health problems (P), which person-related factors (I) are associated with work retention and return to work after sick leave (O)?

## Methods

The Preferred Reporting Items for Systematic Reviews and Meta-Analyses (PRISMA) guidelines were followed as a basis for reporting this systematic review (Moher et al. 2009). This review is registered in the Prospective Register of Systematic Reviews (PROSPERO 2017 registration number CRD42017062459; https://www.crd.york.ac.uk/PROSPERO/).

### Information sources and search strategy

Literature searches were conducted by the first author in the databases PubMed and PsycINFO (MdW). The search strategy had three main elements: health problems, person-related factors and work participation. The main person-related factors of interest that formed the basis of our search strategy were selected by two experts in occupational and insurance medicine. The possible relevance of these factors for work participation was confirmed by a workgroup consisting of three insurance physicians, two occupational physicians and a patient representative. The broad term ‘work participation’ covered concepts such as RTW, sickness absence and current work status. The search strategies used in PubMed and PsycINFO are presented in Online Resource 1.

### Eligibility criteria

This review includes studies published between January 2007 and February 2017 that investigated the association between person-related factors and work participation of employees with health problems. Articles considered eligible for inclusion had to be available in full-text in English or Dutch and had to be published in peer-reviewed journals. We included (non-)randomized controlled trials, cohort studies, cross-sectional studies and qualitative studies. Reference lists of meta-analyses and reviews that were found in our search were examined to identify additional publications, in order not to miss any relevant literature published between 2007 and 2017. Case studies were excluded from this review. Studies in which students, military personnel or veterans with health problems or employees with substance abuse problems were the subjects of the analyses were excluded. We also excluded studies in which disability was the only outcome, or studies in which it was not clear how the person-related factors were measured.

### Study selection

One reviewer selected all relevant studies on the basis of the title and abstract (MdW). The other researchers (HW, CH, MF) each screened the title and abstract of one-third of all studies, so that all studies were independently screened by two reviewers (MdW and HW, MdW and CH or MdW and MF). Subsequently, the full-text articles of potentially relevant studies were reviewed by one reviewer to determine whether they fulfilled all the inclusion criteria (MdW). Additionally, three reviewers screened 10% of the full-text articles (HW, CH, MF). In the case of doubt, eligibility of the study was discussed until consensus was reached. Reasons for exclusion were documented.

### Extraction of data

One reviewer extracted the details and findings of the studies included using a self-developed data-extraction form (MdW). Data-extraction from 30% of the studies was checked by the other three reviewers (HW, CH, MF). Disagreements about the data-extraction were resolved by discussion and consensus. The following details were extracted: number of subjects, age, gender, occupation and health status of subjects, study design, person-related factors of interest, time to follow-up and the main results. To ensure a clear overview of the main results, the coefficients and odds ratios were only noted in the table if they were significant and from multivariate analyses. In addition, we noted *p*-values from significant univariate analyses. Non-significant results were only described in words. We contacted authors when clarification of data was needed.

### Quality assessment

The risk of bias of the studies included was assessed using quality assessment tools developed by the Joanna Briggs Institute (Joanna Briggs Institute 2014). Before the researchers assessed the risk of bias, the Joanna Briggs Institute criteria were discussed between the researchers in order to reduce ambiguity and disagreements between the researchers. One reviewer (MdW) assessed the risk of bias of all studies and the other reviewers (HW, CH, MF) each assessed the risk of bias of 10% of the studies. Disagreements were resolved by discussion and consensus. Each criterion from the quality tools was answered with ‘yes’, ‘no’, ‘unclear’ or ‘not applicable’. For categorizing in studies with high, moderate and low risk of bias, we applied the same classification rules as used in the study by Reilly et al. (2016). Studies which met more than 80% of the criteria were considered as high-quality studies with a low risk of bias. Studies which met 50–80% of the criteria were considered as moderate-quality studies with a moderate risk of bias. Studies which met less than 50% of the criteria were considered as low-quality studies with a high risk of bias. Studies were not excluded on the basis of their risk of bias; however, the risk of bias was taken into account when drawing conclusions in this review.

### Grading the level of evidence

The overall quality of evidence for the association between each person-related factor and work participation was assessed by one reviewer (MdW) using the Grading of Recommendations Assessment, Development and Evaluation (GRADE; Huguet et al. 2013) approach and discussed with the other reviewers (HW, CH, MF). The base level of the quality of evidence for the associations was based on the design and phase of the studies. The factors that were further examined were the risk of bias, inconsistency, indirectness, imprecision and publication bias. The overall quality of evidence for the associations was categorized as high, moderate, low or very low. If possible, a meta-analysis was performed to assess the effects of the person-related factors on work participation.

## Results

### Studies selected

**Fig. 1:**
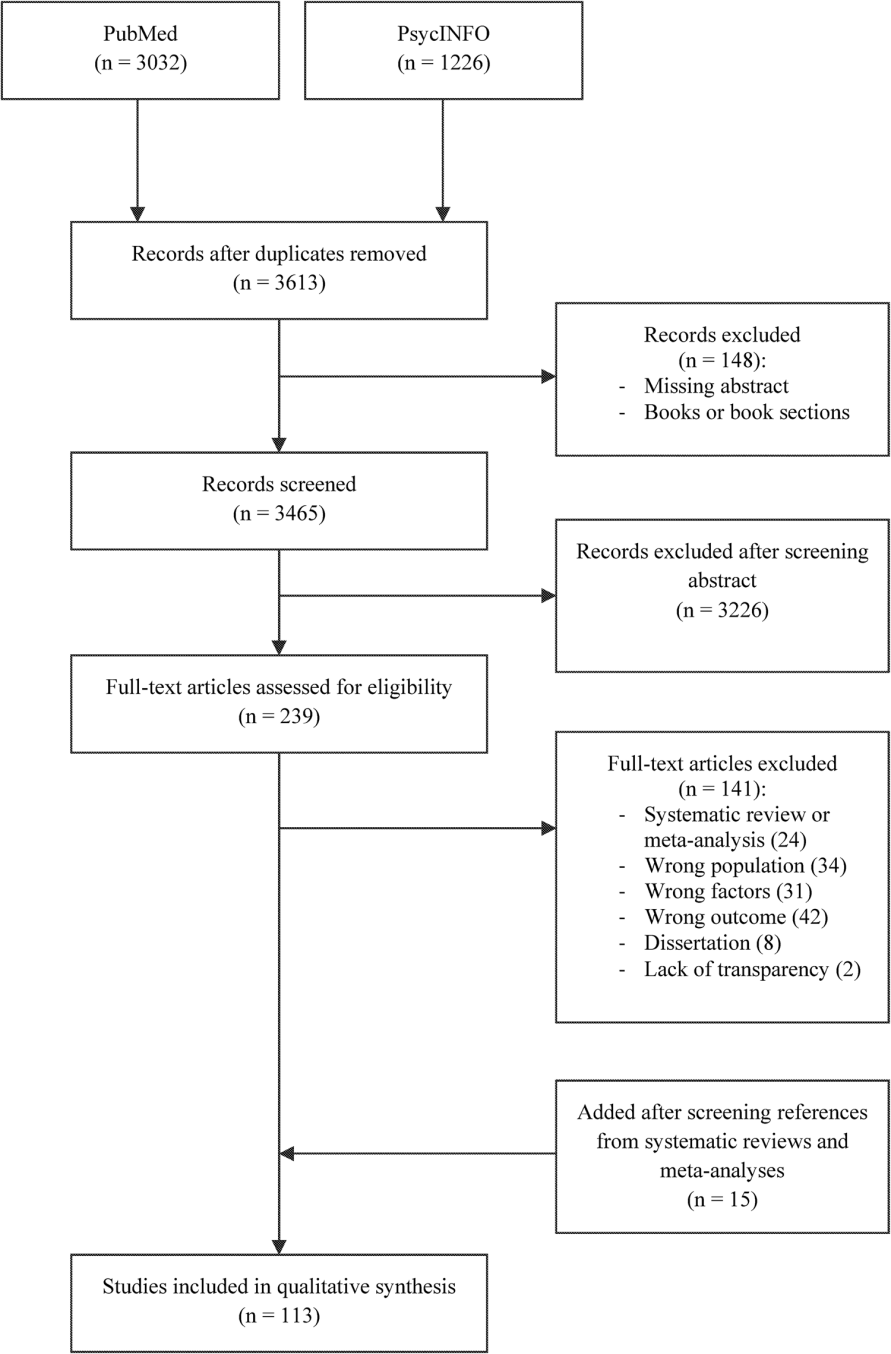
Search flowchart

In total, 3032 studies were found in PubMed and 1226 studies in PsycINFO (Fig. 1). After removing duplicates, studies without abstracts and books or book sections, 3465 studies remained. In total, 3226 studies were excluded after screening the title and abstract. The remaining 239 articles were reviewed on full text. Of these, 117 articles did not meet the inclusion criteria and were thus excluded. The reasons for excluding these articles were: (1) study group did not consist of employees; (2) participants did not have health problems at baseline; (3) factors of interest were not studied; (4) outcome of interest was not studied; (5) study method or results were not (clearly) described; or (6) other study type than (non-)randomized controlled trials, cohort studies, cross-sectional studies, qualitative studies, systematic reviews and meta-analyses. The remaining articles included 24 reviews and meta-analyses. After screening the reference lists of these studies, 15 studies were added, making a total of 113 studies that were included in this review. The characteristics of these studies are presented in the data-extraction tables in Online Resource 2.

### Risk of bias

From the 113 studies included, 68 had a low risk of bias, one study had a high risk of bias and the remaining 44 studies were classified as having a moderate risk of bias. A frequent reason for risk of bias in qualitative studies was that information about the researcher and his possible influence on the study was lacking. Moreover, many cohort studies did not meet the criteria for complete follow-up. Scores on each criterion of the quality assessment tools can be found in Online Resource 3.

### Evidence for the influence of person-related factors on work participation

Results of the multivariate analyses of the quantitative studies that were included in this literature review are summarized in Tables 1 and 2. If no multivariate analyses were performed in a study, conclusions about the association between the person-related factor and work participation were based on the univariate or bivariate analyses that were performed in that study (Denis 2015). Due to the heterogeneity in methods used to measure the person-related factors and outcomes and the heterogeneity in the statistical analyses performed, it was not possible to perform meta-analyses. The quality of evidence for the potential factors associated with work participation as assessed by GRADE is presented in Table 3. Because we used broad terms for the work participation outcomes and used strict inclusion criteria for the participants and the factors measured in the studies, none of the evidence was downgraded for indirectness. In addition, the criteria for publication bias were judged as not applicable, as the large body of evidence made it impossible to come to a conclusion on possible publication bias. Moreover, most of the studies were explorative and the phase of the investigation was already considered as a factor that could downgrade the quality of evidence. The synthesis of evidence led to a rating of moderate evidence for the association between the factors expectations regarding recovery or RTW and perceived health and work participation. The overall quality of evidence for the associations between the person-related factors optimism, catastrophizing, self-efficacy, coping strategies, fear-avoidance beliefs, feelings of control, and perceived work-relatedness of health problems and work participation was rated as low. The evidence for the association between motivation and work participation was rated as very low.

**Table 1:** Results of multivariate analyses of quantitative studies factors expectations, optimism, self-efficacy, motivation, feelings of control, and perceived health

Factor	Positive association +	Negative association −	No association 0
Positive RTW/recovery expectations	**Audhoe et al**. (2012) Besen et al. (2015) **Busch et al**. (2007) **Carriere et al**. (2015a) **Carriere et al**. (2015b) Carstens et al. (2014) **Cowan et al**. (2012) **Du Bois et al**. (2009) **Ekberg et al**. (2015)^**a, b**^ Gross and Battié (2010)^c^ **Hou et al**. (2012) **Hou et al**. (2008) **Jensen et al**. (2013) **Johansson et al**. (2010) **Lindell et al**. (2010**)**^**b**^ Magnussen et al. (2007b) Murgatroyd et al. (2016)^a^ **Opsahl et al**. (2016**)** **Reme et al**. (2009)^**b**^ **Richter et al.** (2011) Rönnberg et al. (2007)^d^ **Sampere et al**. (2012**)** **Sluiter and Frings-Dresen** (2008)^**a**^ **Spector et al.** (2012) Truchon et al. (2012) **Vuistiner et al.** (2015) **Wåhlin et al.** (2012)^**c**^		**Boot et al.** (2008) Coggon et al. (2013) **Ekberg et al.** (2015)^**a**^ Gross and Battié (2010)^c^ Iakova et al. (2012) Murgatroyd et al. (2016)^a^ Nieuwenhuijsen et al. (2013)^d^ **Sluiter and Frings-Dresen** (2008)^**a**^ **Turner et al.** (2008) **Wåhlin et al.** (2012)^**c**^
Optimism	**Hystad and Bye **(2012)^**a, e**^ Lydell et al. (2011)^b, d^		**Hystad and Bye** (2012)^**a, e**^ **Øyeflaten et al.** (2008)
Self-efficacy	Besen et al. (2015) **Brouwer et al**. (2015)^**a, b**^ **Brouwer et al**. (2009)^**a**^ **Brouwer et al**. (2010)^**a**^ **De Vries et al**. (2012b) **Dionne et al.** (2007)^**f**^ **Ekberg et al**. (2015)^**b**^ **Huijs et al.** (2012)^**c**^ Huijs et al. (2017) Lagerveld et al. (2016) Mangels et al. (2011)^g^ Martins (2015)^d^ Nieuwenhuijsen et al. (2013) **Richard et al.** (2011)^**f**^ **Roesler et al**. (2013)^**g**^ **Sarda et al. **(2009)^**h**^ Shaw et al. (2011)^b, g^ Waghorn et al. (2007)	Waynor et al. (2016)^a^	**Brouwer et al**. (2015)^**a**^ **Brouwer et al**. (2009)^**a**^ **Brouwer et al**. (2010)^**a**^ D’Amato and Zijlstra (2010) **Dionne et al.** (2007)^**f**^ **Healey et al.** (2011) **Huijs et al.** (2012)^**c**^ Murphy et al. (2011) **O’Sullivan et al**. (2012) **Øyeflaten et al.** (2008) **Richard et al.** (2011)^**f**^ **Sampere et al.** (2012) **Sarda et al. **(2009)^**h**^ **Strauser et al.** (2010) **Stulemeijer et al**. (2008)^**d**^ **Volker et al.** (2015) **Wåhlin et al.** (2012) Waynor et al. (2016)^a^
Motivation	Awang et al. (2016) **Boyle et al**. (2014)^**d**^ Braathen et al. (2007) Lydell et al. (2011)^b, d^ **Puolakka et al**. (2008)^**f**^ Saperstein et al. (2011)		**Elfving et al**. (2009)^**d**^ **Puolakka et al**. (2008)^**f**^ **Wan Kasim et al.** (2014)
Feelings of control	**Busch et al.** (2007) **Roesler et al.** (2013) **Selander et al. **(2007) **Sluiter and Frings-Dresen **(2008) **Torres et al**. (2009)^**a**^ Truchon et al. (2010)^b^ **Vlasveld et al**. (2013)^**b**^		**Boot et al.** (2008) **Ekberg et al.** (2015) Karoly et al. (2013) Krause et al. (2013)^d^ Murphy et al. (2011) **Richard et al.** (2011) **Torres et al**. (2009)^**a**^ **Volker et al.** (2015)
Perceived health	**Audhoe et al**. (2012)^**b, g**^ **Boot et al**. (2014)^**d**^ Boot et al. (2011) Dawson et al. (2011)^i^ **Dionne et al.** (2007)^**f**^ **Dyster-Aas et al**. (2007)^**d**^ **Ekberg et al**. (2015)^**b**^ **Grøvle et al.** (2013)^**f**^ **He et al.** (2010) Iakova et al. (2012)^a^ Morrison et al. (2016)^d^ Murgatroyd et al. (2016) **Nielsen et al. **(2012) **Ramel et al**. (2013)^**d**^ **Sampere et al.** (2012)^**c, e,f**^ **Sivertsen et al.** (2013) **Vuistiner et al.** (2015)		**Chen et al.** (2012) **De Vries et al**. (2012b)^**d**^ **Dionne et al.** (2007)^**f**^ **Elfving et al**. (2009)^**d**^ **Grøvle et al**. (2013)^**f**^ Hansen et al. (2009) Iakova et al. (2012)^a^ **Jensen et al.** (2013) **Lindell et al**. (2010**)** **Richter et al**. (2011**)** **Sampere et al.** (2012)^**c, e, f**^

Bold indicates studies with a low risk of bias

^a^Depends on the form/subscale of the factor

^b^Not for every moment on which the outcome is measured

^c^Depends on the type of disorder of the participant

^d^Outcomes from univariate analysis

^e^Depends on the gender of the participant

^f^Depends on the form of work participation

^g^Not for every moment on which the factor is measured

^h^Depends on the country where the participant lives

^i^Outcomes from bivariate analysis

**Table 2:** Results of multivariate analyses of quantitative studies with factors coping strategies, fear-avoidance, work-relatedness and catastrophizing

Factor	Positive association +	Negative association −	No association 0
Coping strategies	**Huijs et al. **(2012)^**a, c**^ Karoly et al. (2013) **Øyeflaten et al.** (2008)^**a**^	**Arwert et al.** (2017)^**a**^ Dawson et al. (2011)^a^ **Grytten et al**. (2017)^**a, g**^ Iakova et al. (2012) Karoly et al. (2013)^d^ **Norlund et al. **(2011) Strober and Arnett (2016)^a^	**Arwert et al.** (2017)^**a**^ Dawson et al. (2011)^a, d^ **De Vries et al**. (2012b) **Grytten et al**. (2017)^**a, g**^ **Heymans et al.** (2009) **Huijs et al.** (2012)^**a, c**^ **Luk et al**. (2010)^**d**^ **Øyeflaten et al.** (2008)^**a**^ Øyeflaten et al. (2016) Strober and Arnett (2016)^a^ Truchon et al. (2010)
Fear-avoidance beliefs		Coggon et al. (2013)^i^ Dawson et al. (2011)^i^ **De Vries et al**. (2012b)^i^ **Dionne et al**. (2007)^**e, g, i, j**^ **Du Bois et al.** (2009)^**i**^ **Dyster-Aas et al**. (2007)^**d, k**^ **Grøvle et al. **(2013)^**d**, **g, h,i**^ **Heymans et al**. (2009)^**k**^ **Heymans et al**. (2007)^**k**^ **Kovacs et al.** (2007)^**j, k**^ Magnussen et al. (2007b)^j^ Mannion et al. (2009)^j^ **Morris and Watson** (2011)^**j**^ **Opsahl et al**. (2016)^**h, j**^ **Øyeflaten et al**. (2008)^**j**^ Øyeflaten et al. (2016)^j^ Truchon et al. (2012)^j^	Besen et al. (2015)^j^ **Carriere et al**. (2015a)^**i**^ **Dionne et al**. (2007)^**e**, **j**^ **Du Bois et al. **(2009)^**j**^ **Elfving et al**. (2009)^**d**^ **Grøvle et al.** (2013)^**d, g**, i^ **Heymans et al**. (2009)^**i**^ **Jensen et al**. (2013)^**k**^ Karels et al. (2010)^j^ **Kovacs et al**. (2007)^**i**^ Magnussen et al. (2007b)^i^ Mannion et al. (2009)^i^ **Morris and Watson** (2011)^**d, i**^ **Øyeflaten et al**. (2008)^**i**^ Øyeflaten et al. (2016)^d, i^ **Poulain et al**. (2010)^**i, j, k**^ **Richter et al**. (2011)^**i**^ **Spector et al**. (2012)^**j**^ **Steenstra et al**. (2010)^**d, i, j**^ **Turner et al**. (2008)^**j**^
Perceived work-relatedness		**Jensen et al.** (2013) Karels et al. (2010) **Kuijer et al.** (2016) **Sampere et al.** (2012)^**c, e**^	Coggon et al. (2013) Dawson et al. (2011) **Sampere et al.** (2012)^**c, e**^ **Turner et al**. (2008)^**d**^
Catastrophizing		**Adams et al.** (2017) **Carriere et al.** (2015a) **Cowan et al**. (2012)^**f, g**^ **De Vries et al**. (2012b) Karoly et al. (2013) **Lindell et al**. (2010)^**b**^ Wijnhoven et al. (2007)	Besen et al. (2015) **Cowan et al. **(2012)^**f, g**^ Dawson et al. (2011) Karels et al. (2010) Mannion et al. (2009) **Morris and Watson** (2011)^**d**^ **Sarda et al. **(2009) **Spector et al.** (2012) **Turner et al.** (2008)

Bold indicates studies with a low risk of bias

^a^Depends on the form/subscale of the factor

^b^Not for every moment on which the outcome is measured

^c^Depends on the type of disorder of the participant

^d^Outcomes from univariate analysis

^e^Depends on the gender of the participant

^f^Depends on the job of the participant

^g^Depends on the form of work participation

^h^  Outcomes from bivariate analysis

^i^Fear-avoidance beliefs for movement/physical activity

^j^Fear-avoidance beliefs for work

^k^Total fear-avoidance

**Table 3:** GRADE assessment of selected potential factors associated with work participation

Factor	Study design	Study phase	Quality assessment					Summary of findings			
			Risk of bias	Inconsistency	Indirectness^a^	Imprecision^c^	Publication bias^b^	Effect			Overall quality
								+	−	0	
Positive expectations regarding recovery or RTW	RCT: 1 PCS/RCS: 28 CSS: 3	Confirmative: 10 Explorative: 22	✓	✓	✓	✓	n.a	27	0	10	Moderate +++
Optimism	RCT: 0 PCS/RCS: 3 CSS: 0	Confirmative: 1 Explorative: 2	✓	✗	✓	n.a.^d^	n.a	2	0	2	Low ++
Self-efficacy	RCT: 0 PCS/RCS: 23 CSS: 6	Confirmative: 11 Explorative: 18	✓	✗	✓	✓	n.a	18	1	18	Low ++
Motivation	RCT: 0 PCS/RCS: 3 CSS: 4 Non-RCT: 1	Confirmative: 1 Explorative:7	✗	✓	✓	✓	n.a	6	0	3	Very low +
Feelings of control	RCT: 0 PCS/RCS: 9 CSS: 5	Confirmative: 5 Explorative: 9	✓	✗	✓	✓	n.a	7	0	8	Low ++
Perceived health	RCT: 0 PCS/RCS: 18 CSS: 6	Confirmative: 2 Explorative: 22	✓	✓	✓	✓	n.a	17	0	11	Moderate +++
Coping strategies	RCT: 0 PCS/RCS: 9 CSS: 5	Confirmative: 3 Explorative: 11	✓	✗	✓	✓	n.a	3	7	11	Low ++
Fear-avoidance beliefs	RCT: 1 PCS/RCS: 19 CSS: 7	Confirmative: 9 Explorative: 18	✓	✗	✓	✓	n.a	0	17	20	Low ++
Perceived work-relatedness	RCT: 0 PCS/RCS: 5 CSS: 2	Confirmative: 0 Explorative: 7	✓	✗	✓	✓	n.a	0	4	4	Low ++
Catastrophizing	RCT: 0 PCS/RCS: 8 CSS: 7	Confirmative: 5 Explorative: 10	✓	✗	✓	✓	n.a	0	7	9	Low ++

*RCT* randomized controlled trial, *PCS* prospective cohort study, *RCS* retrospective cohort study, *CSS* cross-sectional study

^a^The quality of evidence was not downgraded for indirectness due to the broad terms for work participations and the strict inclusion criteria for the participants and factors used for this study

^b^The quality of evidence was not downgraded for publication bias because of the large body of evidence and because most of the studies were explorative studies and phase of investigation was already taken into account as a factor that could downgrade the overall quality of evidence

^c^Studies which did not report confidence intervals for both significant and non-significant results, were not taken into account when deciding when to downgrade for imprecision

^d^The quality of evidence was not downgraded for imprecision because none of the studies in which the effect of pessimism or optimism was non-significant reported confidence intervals

#### Expectations regarding recovery or RTW

In total, 32 quantitative studies investigated the association between expectations regarding recovery or RTW and work participation (Table 1: Audhoe et al. 2012; Besen et al. 2015; Boot et al. 2008; Busch et al. 2007; Carriere et al. 2015a, b; Carstens et al. 2014; Coggon et al. 2013; Cowan et al. 2012; Du Bois et al. 2009; Ekberg et al. 2015; Gross and Battié 2010; Hou et al. 2008, 2012; Iakova et al. 2012; Jensen et al. 2013; Johansson et al. 2010; Lindell et al. 2010; Magnussen et al. 2007b; Murgatroyd et al. 2016; Nieuwenhuijsen et al. 2013; Opsahl et al. 2016; Reme et al. 2009; Richter et al. 2011; Rönnberg et al. 2007; Sampere et al. 2012; Sluiter and Frings-Dresen 2008; Spector et al. 2012; Truchon et al. 2012; Turner et al. 2008; Vuistiner et al. 2015; Wåhlin et al. 2012). The majority of these studies found evidence of a positive association, which suggests that having positive expectations about one’s recovery or chances of RTW has a positive effect on work participation for employees with health problems (Audhoe et al. 2012; Besen et al. 2015; Busch et al. 2007; Carriere et al. 2015a, b; Carstens et al. 2014; Cowan et al. 2012; Du Bois et al. 2009; Ekberg et al. 2015; Gross and Battié 2010; Hou et al. 2008, 2012; Jensen et al. 2013; Johansson et al. 2010; Lindell et al. 2010; Magnussen et al. 2007b; Murgatroyd et al. 2016; Opsahl et al. 2016; Reme et al. 2009; Richter et al. 2011; Rönnberg et al. 2007; Sampere et al. 2012; Sluiter and Frings-Dresen 2008; Spector et al. 2012; Truchon et al. 2012; Vuistiner et al. 2015; Wåhlin et al. 2012). However, some of these studies indicated that the effect was dependent on the subgroup of participants or the form of expectations (Ekberg et al. 2015; Gross and Battié 2010; Murgatroyd et al. 2016; Sluiter and Frings-Dresen 2008; Wåhlin et al. 2012). For example, in a study by Ekberg et al. (2015), positive recovery expectations were associated with early RTW, but RTW expectations were not. Only four studies did not find any association between expectations and work participation in multivariate analyses (Boot et al. 2008; Coggon et al. 2013; Iakova et al. 2012; Turner et al. 2008). There were no qualitative studies which suggested a positive association between these expectations and work participation. The overall quality of evidence for the effect of expectations regarding recovery or RTW on work participation was moderate. It was downgraded because all evidence came from explorative studies.

#### Optimism

Being optimistic or pessimistic was the least investigated person-related factor addressed in the studies found in this systematic review. Three quantitative studies investigated the influence of optimism or pessimism (Table 1: Hystad and Bye 2012; Lydell et al. 2011; Øyeflaten et al. 2008). One quantitative study reported a negative effect of pessimism on RTW, but did not find any effect of optimism (Hystad and Bye 2012). This was in contrast to a study by Øyeflaten et al. (2008) which reported that being pessimistic about oneself and the future had no significant effect on RTW, and to a study by Lydell et al. (2011), which found support for a positive effect of optimism on RTW. There were three qualitative studies in which it was mentioned that being optimistic was important for work participation (De Vries et al. 2011; Ellingsen and Aas 2009; Lundqvist and Samuelsson 2012). In summary, the majority of the studies suggest a positive association between optimism and work participation in employees with health problems. As most evidence came from explorative studies and because of inconsistency in study results, the overall quality of evidence was rated as low.

#### Self-efficacy

The association between self-efficacy and work participation was investigated in 29 quantitative studies (Table 1: Besen et al. 2015; Brouwer et al. 2009, 2010, 2015; D’Amato and Zijlstra 2010; De Vries et al. 2012b; Dionne et al. 2007; Ekberg et al. 2015; Healey et al. 2011; Huijs et al. 2012, 2017; Lagerveld et al. 2016; Mangels et al. 2011; Martins 2015; Murphy et al. 2011; Nieuwenhuijsen et al. 2013; O’Sullivan et al. 2012; Øyeflaten et al. 2008; Richard et al. 2011; Roesler et al. 2013; Sampere et al. 2012; Sarda et al. 2009; Shaw et al. 2011; Strauser et al. 2010; Stulemeijer et al. 2008; Volker et al. 2015; Waghorn et al. 2007; Wåhlin et al. 2012; Waynor et al. 2016). Eleven studies found a positive association between self-efficacy and work participation (Besen et al. 2015; De Vries et al. 2012b; Ekberg et al. 2015; Huijs et al. 2017; Lagerveld et al. 2016; Mangels et al. 2011; Martins 2015; Nieuwenhuijsen et al. 2013; Roesler et al. 2013; Shaw et al. 2011; Waghorn et al. 2007). Two studies found evidence of a positive association between self-efficacy and work participation for only some specific subgroups (Huijs et al. 2012; Sarda et al. 2009). The results of three other studies suggest that the association depends on the form of self-efficacy (Brouwer et al. 2015, 2009, 2010). In summary, the majority of the results suggest that having more self-efficacy is associated with more work participation in employees with health problems. Six qualitative studies supported these results (De Vries et al. 2011; Dunn et al. 2010; Hartke et al. 2011; Lundqvist and Samuelsson 2012; Magnussen et al. 2007a; Tamminga et al. 2012). However, some quantitative studies did not find evidence of an association between self-efficacy and work participation (D’Amato and Zijlstra 2010; Healey et al. 2011; Murphy et al. 2011; O’Sullivan et al. 2012; Øyeflaten et al. 2008; Sampere et al. 2012; Strauser et al. 2010; Stulemeijer et al. 2008; Volker et al. 2015; Wåhlin et al. 2012). One study even found a negative association between work-related social skills self-efficacy and current employment status (Waynor et al. 2016). As there was serious inconsistency in study results, the overall quality of evidence was downgraded to low.

#### Motivation

Of the eight quantitative studies which investigated the association between motivation and work participation (Table 1: Awang et al. 2016; Boyle et al. 2014; Braathen et al. 2007; Elving et al. 2009; Lydell et al. 2011; Puolakka et al. 2008; Saperstein et al. 2011; Wan Kasim et al. 2014), five found a positive association (Awang et al. 2016; Boyle et al. 2014; Braathen et al. 2007; Lydell et al. 2011; Saperstein et al. 2011). One additional quantitative study only found an influence of motivation for some forms of work participation (Puolakka et al. 2008). This study by Puolakka et al. (2008) indicated that motivation to work was associated with fewer days off work, but not with permanent work disability. Two studies with a low risk of bias did not find any association between motivation and work participation (Elfving et al. 2009; Wan Kasim et al. 2014). Seven qualitative studies addressed the possible positive influence of motivation, which suggests that employees with health problems who are motivated will have higher levels of work participation (Åhrberg et al. 2010; De Vries et al. 2011; Dekkers-Sánchez et al. 2010; Dunn et al. 2010; Hartke et al. 2011; Van Velzen et al. 2011; Wilbanks and Ivankova 2015). However, the overall quality of the quantitative evidence for this factor was downgraded to very low because evidence primarily came from explorative studies with serious risk of bias.

#### Feelings of control

There were 14 quantitative studies which addressed the possible positive association between feelings of control and work participation (Table 1: Boot et al. 2008; Busch et al. 2007; Ekberg et al. 2015; Karoly et al. 2013; Krause et al. 2013; Murphy et al. 2011; Richard et al. 2011; Roesler et al. 2013; Selander et al. 2007; Sluiter and Frings-Dresen 2008; Torres et al. 2009; Truchon et al. 2010; Vlasveld et al. 2013; Volker et al. 2015). The results of six studies indicated that the feeling of having more control is associated with more work participation (Busch et al. 2007; Roesler et al. 2013; Selander et al. 2007; Sluiter and Frings-Dresen 2008; Truchon et al. 2010; Vlasveld et al. 2013). These results were supported by one qualitative study by Dionne et al. (2013), in which it was reported that participants who did not RTW considered that their return depended more on factors related to their environment than on personal factors. However, one quantitative study only found evidence on some specific forms of control but not others (Torres et al. 2009). For example, in this study, having the feeling that one controls one’s own pain was not associated with RTW, but believing that control of pain is a chance outcome decreased the likelihood of RTW (Torres et al. 2009). Seven studies found no evidence of an association between feelings of control and work participation at all (Boot et al. 2008; Ekberg et al. 2015; Karoly et al. 2013; Krause et al. 2013; Murphy et al. 2011; Richard et al. 2011; Volker et al. 2015). The overall quality of evidence derived from this review was low and was downgraded for serious inconsistency.

#### Perceived health

Twenty-four quantitative studies addressed the possible influence of perceived health on work participation (Table 1: Audhoe et al. 2012; Boot et al. 2011, 2014; Chen et al. 2012; Dawson et al. 2011; De Vries et al. 2012b; Dionne et al. 2007; Dyster-Aas et al. 2007; Ekberg et al. 2015; Elfving et al. 2009; Grøvle et al. 2013; Hansen et al. 2009; He et al. 2010; Iakova et al. 2012; Jensen et al. 2013; Lindell et al. 2010; Morrison et al. 2016; Murgatroyd et al. 2016; Nielsen et al. 2012; Ramel et al. 2013; Richter et al. 2011; Sampere et al. 2012; Sivertsen et al. 2013; Vuistiner et al. 2015). Seven studies found no association at all between the factor and work participation (Chen et al. 2012; De Vries et al. 2012b; Elfving et al. 2009; Hansen et al. 2009; Jensen et al. 2013; Lindell et al. 2010; Richter et al. 2011). However, the majority of the studies found that being positive about one’s general health was positively associated with work participation (Audhoe et al. 2012; Boot et al. 2011, 2014; Dawson et al. 2011; Dionne et al. 2007; Dyster-Aas et al. 2007; Ekberg et al. 2015; He et al. 2010; Morrison et al. 2016; Murgatroyd et al. 2016; Nielsen et al. 2012; Ramel et al. 2013; Sivertsen et al. 2013; Vuistiner et al. 2015). There were three studies in which the results indicated that this association was dependent on the form of work participation (Dionne et al. 2007; Grøvle et al. 2013; Sampere et al. 2012). For example, the results of the study by Grøvle et al. (2013) suggested that perceived health was positively associated with the likelihood of RTW, but not with number of days until sustained RTW. The results of a study by Iakova et al. (2012) indicated that improvement in general health was associated with a higher likelihood of RTW, but general health at baseline and physical health were not. There were no qualitative studies which addressed the association between perceived health and work participation. The quality of evidence was rated as moderate.

#### Coping strategies

In total, 14 quantitative studies investigated the association between different coping strategies and work participation (Table 2: Arwert et al. 2017; Dawson et al. 2011; De Vries et al. 2012b; Grytten et al. 2017; Heymans et al. 2009; Huijs et al. 2012; Iakova et al. 2012; Karoly et al. 2013; Luk et al. 2010; Norlund et al. 2011; Øyeflaten et al. 2008, 2016; Strober and Arnett 2016; Truchon et al. 2010). Nine studies found an effect of some coping strategies (Arwert et al. 2017; Dawson et al. 2011; Grytten et al. 2017; Huijs et al. 2012; Iakova et al. 2012; Karoly et al. 2013; Norlund et al. 2011; Øyeflaten et al. 2008; Strober and Arnett 2016). The results of these studies indicated that some coping strategies, such as active problem-solving (Huijs et al. 2012), could increase the chance of work participation in sick employees, while other coping strategies, such as passive coping (Dawson et al. 2011) and avoidance coping (Iakova et al. 2012), could decrease the chance of work participation. However, five of the quantitative studies did not find any evidence of the effect of coping strategies (De Vries et al. 2012b; Heymans et al. 2009; Luk et al. 2010; Øyeflaten et al. 2016; Truchon et al. 2010). Six qualitative studies addressed the importance of different coping strategies for work participation (Becker et al. 2007; Dekkers-Sánchez et al. 2010; De Vries et al. 2011; Hartke et al. 2011; Lundqvist and Samuelsson 2012; Tamminga et al. 2012). The overall quality of evidence derived from this review was downgraded to low because most of the studies were explorative and because of serious inconsistency in study results.

#### Fear-avoidance beliefs

In total, 27 studies addressed the possible association between fear-avoidance beliefs and work participation (Table 2: Besen et al. 2015; Carriere et al. 2015a; Coggon et al. 2013; Dawson et al. 2011; De Vries et al. 2012b; Dionne et al. 2007; Du Bois et al. 2009; Dyster-Aas et al. 2007; Elfving et al. 2009; Grøvle et al. 2013; Heymans et al. 2007, 2009; Jensen et al. 2013; Karels et al. 2010; Kovacs et al. 2007; Magnussen et al. 2007b; Mannion et al. 2009; Morris and Watson 2011; Opsahl et al. 2016; Øyeflaten et al. 2008, 2016; Poulain et al. 2010; Richter et al. 2011; Spector et al. 2012; Steenstra et al. 2010; Truchon et al. 2012; Turner et al. 2008). Most of the studies made a distinction between fear-avoidance beliefs for movement or physical activity and fear-avoidance beliefs for work. Six studies did find an association between fear-avoidance beliefs for work and work participation, but did not find an association between fear-avoidance beliefs for physical activity or movement and work participation (Kovacs et al. 2007; Magnussen et al. 2007b; Mannion et al. 2009; Morris and Watson 2011; Øyeflaten et al. 2008, 2016). However, Du Bois et al. (2009) reported that fear-avoidance beliefs for movement, rather than fear-avoidance beliefs for work were associated with a higher chance of not returning to work. Three studies which only studied fear-avoidance for movement or physical activity found negative associations between this factor and work participation (Coggon et al. 2013; Dawson et al. 2011; De Vries et al. 2012b). Two studies which only investigated the association between fear-avoidance for work and work participation, also found negative associations (Opsahl et al. 2016; Truchon et al. 2012). Three studies found a negative association between general fear-avoidance and work participation (Dyster-Aas et al. 2007; Heymans et al. 2007, 2009). Studies by Dionne et al. (2007) and Grøvle et al. (2013) indicated that the effect of fear-avoidance was dependent on how work participation is measured. For example, the results of the study by Grøvle et al. (2013) suggested that fear-avoidance for movement is associated with the likelihood of RTW within two years, but not with number of days until sustained RTW. Ten studies did not find any association between fear-avoidance beliefs and work participation (Besen et al. 2015; Carriere et al. 2015a; Elfving et al. 2009; Jensen et al. 2013; Karels et al. 2010; Poulain et al. 2010; Richter et al. 2011; Spector et al. 2012; Steenstra et al. 2010; Turner et al. 2008). There were no qualitative studies which addressed this association. However, overall, the majority of the studies which investigated fear-avoidance beliefs, found a negative association between fear-avoidance and work participation. Because most of these studies were explorative and because there was serious inconsistency in study results, the overall quality of evidence was downgraded to low.

#### Perceived work-relatedness

Of the limited number of studies that addressed the relationship between perceiving the health problem as work-related and work participation, three studies did not find any association between this factor and work participation (Coggon et al. 2013; Dawson et al. 2011; Turner et al. 2008), while three studies found a negative association (Table 2: Jensen et al. 2013; Karels et al. 2010; Kuijer et al. 2016). These latter studies suggest that perceiving the health problem as work-related has a negative association with work participation in employees with health problems. Findings from an additional study, by Sampere et al. (2012), supported this negative association, but only for women and only for employees with mental disorders. There were no qualitative studies which addressed this association. As all of the studies which investigated this association were explorative and because there was serious inconsistency, the overall quality of evidence was downgraded to low.

#### Catastrophizing

Fifteen quantitative studies examined the association between catastrophizing and work participation (Table 2: Adams et al. 2017; Besen et al. 2015; Carriere et al. 2015a; Cowan et al. 2012; Dawson et al. 2011; De Vries et al. 2012b; Karels et al. 2010; Karoly et al. 2013; Lindell et al. 2010; Mannion et al. 2009; Morris and Watson 2011; Sarda et al. 2009; Spector et al. 2012; Turner et al. 2008; Wijnhoven et al. 2007). Eight quantitative studies in this review did not find an effect of catastrophizing on work status or sickness absence (Besen et al. 2015; Dawson et al. 2011; Karels et al. 2010; Mannion et al. 2009; Morris and Watson 2011; Sarda et al. 2009; Spector et al. 2012; Turner et al. 2008). No qualitative studies reported the negative influence of catastrophizing on work participation. However, six quantitative studies (Adams et al. 2017; Carriere et al. 2015a; De Vries et al. 2012b; Karoly et al. 2013; Lindell et al. 2010; Wijnhoven et al. 2007), including four studies with a low risk of bias (Adams et al. 2017; Carriere et al. 2015a; De Vries et al. 2012b; Lindell et al. 2010), found a negative association between catastrophizing and work participation. One quantitative study found a negative association between catastrophizing and return to modified work for some subgroups (Cowan et al. 2012). The evidence suggests that catastrophizing is negatively associated with work participation. Because most of the evidence came from explorative studies and because there was serious inconsistency, its overall quality was downgraded to low.

## Discussion

This systematic review of 113 studies identified the association between ten selected person-related factors and work participation of employees with health problems. The factors positively associated with work participation were positive expectations regarding recovery or RTW, optimism, self-efficacy, motivation, feelings of control, and perceived health. The factors negatively associated with work participation were fear-avoidance beliefs, perceived work-relatedness of the health problem and catastrophizing. Coping strategies had both positive and negative associations with work participation.

The synthesis of evidence showed that we can be moderately confident that positive expectations regarding recovery or RTW and better self-perceived health lead to a higher level of work participation in employees with health problems. This possible association between these expectations and work participation is in line with the findings of a review by Iles et al. (2009), in which recovery expectations in employees with low back pain were a strong predictor of work outcome. Our finding on the association between self-perceived health and work participation is supported by the results of a review by Lidal et al. (2007), in which poor state of health was one of the most frequent self-reported barriers to employment in employees with spinal cord injury.

For the person-related factors optimism, catastrophizing, self-efficacy, coping strategies, fear-avoidance beliefs, feelings of control, and perceived work-relatedness of health problems, the quality of evidence for an association with work participation was rather low. Nevertheless, the results of this review suggest that fear-avoidance beliefs, perceived work-relatedness of health problems and catastrophizing are negatively associated with work participation. Optimism, self-efficacy and feelings of control seem to lead to more work participation. According to the results of our review, different coping strategies can have a positive or a negative effect on work participation.

The results of our review of these factors are consistent with the results of a Delphi study by Peters et al. (2017), which indicate that researchers and clinicians in the field of work disability or RTW identify most of these factors (catastrophizing, self-efficacy, coping strategies, fear-avoidance beliefs and feelings of control) as affecting work participation. However, the results of the current review partly stand in contrast to the results of a review by De Vries et al. (2012a), in which catastrophizing had no association with remaining at work for employees at all. However, that review only included three cross-sectional studies on employees with chronic non-specific musculoskeletal pain, including two of low quality, which might explain this contradictory finding.

Studies conducted by Achterberg et al. (2012) and Peters et al. (2017) found that insurance physicians and experts identified motivation as the most important person-related factor for work participation. The results of the qualitative studies included in the current review are in line with this (Åhrberg et al. 2010; De Vries et al. 2011; Dekkers-Sánchez et al. 2010; Dunn et al. 2010; Hartke et al. 2011; Van Velzen et al. 2011; Wilbanks and Ivankova 2015). Surprisingly, the current review found a very low quality of quantitative evidence for an association between motivation and work participation. The results of a review by Faber et al. (2016) indicate that motivation consists of seven underlying aspects, including intrinsic motivation, expectations and self-efficacy. If researchers choose to study individual aspects of motivation rather than overall motivation, this could explain why we did not find many studies addressing the association between overall motivation and work participation. Moreover, when researchers choose to study the effects of factors such as self-efficacy and expectations alongside motivation, the overall effect of motivation could be underestimated due to the association with these other factors. These reasons could explain why we found very low evidence for an association between motivation and work participation.

### Strengths and limitations of the current review

This systematic review studied the association between a set of selected person-related factors and work participation and was not limited to specific diseases or disorders; this makes the results of this study generalizable to various health problems. A key methodological strength of this review is that the articles were screened and assessed by multiple independent reviewers, explicitly to avoid bias. In addition, the quality of the studies as assessed by the assessment tools of the Joanna Briggs Institute (2014), was considered when interpreting the results of this review. The benefit of using these tools is that, although they are adapted to different study designs, the criteria on which the risk of bias is assessed are comparable between the different tools. A final strength of our study is that when assessing the level of evidence for possible associations, we used the GRADE framework for prognostic factor research (Huguet et al. 2013) to prevent errors in judgement.

Despite methodological strengths, there were also some constraints in the methodology of our review. We included 113 studies which had different ways of defining and measuring the person-related factors, which raises uncertainties in the interpretation of our findings. Besides, we included studies with participants with different diseases and disorders and participants with different occupations. At first sight, the diversity in participants improves the generalizability of our findings. However, it is possible that the influence of some of the studied factors on work participation differs across participants with different diseases and disorders or differs across occupations, which may also raise uncertainties in the interpretation of our findings. Moreover, due to heterogeneity of measurements of factors and outcomes, and heterogeneity in the statistical analyses performed in these studies, it was not possible to perform a meta-analysis. Furthermore, not every study controlled for the same variables in their analysis, and therefore there may have been hidden variables which may have influenced the outcomes.

### Implications for practice and future research

We suggest that in addition to health-related factors and environmental factors, person-related factors should be considered by occupational physicians and insurance physicians when they diagnose, evaluate or provide treatment to employees. In particular, the factors perceived health and expectations regarding recovery or RTW may have significant influence on work participation and, therefore, they should be considered by occupational and insurance physicians in their efforts to improve work participation of employees with health problems.

Although the results of this review suggest that person-related factors are associated with work participation, the quality of evidence for the involvement of some of these factors was low or very low. Therefore, more research is needed to improve the quality of evidence for the involvement of these factors. Future research should also focus on how physicians might gain more insight into these different cognitions and perceptions of employees. This might assist in the identification of barriers to RTW or barriers to remaining at work for employees with health problems. Finally, research will be needed to determine whether the use of information about person-related factors by physicians improves work participation of employees with health problems and leads to a better quality of care.

## Electronic supplementary material

Below is the link to the electronic supplementary material.

Supplementary material 1 (PDF 43 KB)

Supplementary material 2 (PDF 540 KB)

Supplementary material 3 (PDF 362 KB)
