# Supplementary material for: Person-related factors associated with work participation in employees with health problems: a systematic review
Source: Int Arch Occup Environ Health. 2018 Apr 26;91(5):497–512. doi: 10.1007/s00420-018-1308-5 (PMC6002456; doi:10.1007/s00420-018-1308-5)
Supplement: Supplementary file 1 — Supplementary material 1 (PDF 43 KB) [file 420_2018_1308_MOESM1_ESM.pdf]

Person-related factors associated with work participation in employees with health problems: a systematic review

M. de Wit, H. Wind, C. T. J. Hulshof, M. H. W. Frings-Dresen

Department Coronel Institute of Occupational Health, Academic Medical Center, University of Amsterdam, Amsterdam Public Health research institute, Amsterdam, the Netherlands

Correspondence to: Mariska de Wit, Department Coronel Institute of Occupational Health, Academic Medical Center, University of Amsterdam, Amsterdam Public Health research institute, PO Box 22700, NL-1100 DE Amsterdam, The Netherlands; m.e.dewit@amc.uva.nl; +31(0)20-5665341

Journal: International Archives of Occupational and Environmental Health

Online Resource 1

Online Resource 1

PubMed search strategy

Search	Query
#1 Population of interest	"chronic disease"[Mesh] OR chronic disease*[tw] OR chronic health[tw] OR chronic condition*[tw] OR long-term sickness[tw] OR long-term sick[tw] OR chronic patients[tw] OR disorder[tw] OR disorders[tw] OR disability[tw] OR disabilities[tw] OR injury[tw] OR injuries[tw]
#2 Person-related factors	"diagnostic self evaluation"[Mesh] OR self evaluation[tw] OR subjective health complaints[tw] OR self-appraisal[tw] OR health concerns[tw] OR perceived health[tw] OR illness perceptions[tw] OR patient beliefs[tw] OR patient perceptions[tw] OR perceived severity[tw] OR disability perceptions[tw] OR pain beliefs[tw] OR perceived disability[tw] OR perceived illness[tw] OR perceived impairment[tw] OR beliefs about illness[tw] OR illness beliefs[tw] OR illness representations[tw] OR illness cognitions[tw] OR illness identity[tw] OR psychological factors[tw] OR Motivation[Mesh:NoExp] OR motivation[tw] OR motivated[tw] OR unmotivated[tw] OR willingness to work[tw] OR meaning of work[tw] OR employee motivation[tw] OR work attitudes[tw] OR work motives[tw] OR work reasons[tw] OR work drive[tw] OR rehabilitation expectation*[tw] OR patient expectation*[tw] OR prognostic expectation*[tw] OR improvement expectation*[tw] OR recovery expectation*[tw] OR return to work expectation*[tw] OR RTW expectation*[tw] OR negative expectation*[tw] OR positive expectation*[tw] OR optimism[Mesh] OR optimism[tw] OR pessimism[Mesh] OR pessimism[tw] OR expected outcome[tw] OR perceived curability[tw] OR perceived work ability[tw] OR irrational cognitions[tw] OR irrational beliefs[tw] OR irrational thoughts[tw] OR irrational feelings[tw] OR irrationality[tw] OR Catastrophization[Mesh] OR catastrophization[tw] OR catastrophizing[tw] OR cognitive insufficiency[tw] OR negative perceptions[tw] OR negative orientation[tw] OR negative thoughts[tw] OR Shared decision making[tw] OR involvement in decision making[tw] OR participation in decision making[tw] OR patient participation[Mesh] OR patient participation[tw] OR client participation[tw] OR informed decision making[tw] OR "patient preference"[Mesh] OR patient preference*[tw] OR "internal-external control"[Mesh:NoExp] OR internal-external control[tw] OR control orientation[tw] OR control beliefs[tw] OR perceptions of control[tw] OR illness controllability[tw] OR perceived cause*[tw] OR external orientation[tw] OR internal orientation[tw] OR work-related*[tw] OR "Adaptation, Psychological"[Mesh] OR psychological adaption[tw] OR coping[tw] OR fear-avoidance[tw] OR adaptive response[tw] OR avoidance behavior[tw] OR cognitive reappraisal[tw] OR "Self concept"[Mesh] OR self-concept[tw] OR self-efficacy[tw] OR self-confidence[tw] OR self-esteem[tw] OR perceived ability[tw] OR self-image[tw] OR perceived competence[tw]
#3 Work participation outcome	"return to work"[Mesh] OR (return to[tw] AND work[tw]) OR RTW[tw] OR returning to work[tw] OR back to work[tw] OR unemployment[Mesh] OR unemployment[tw] OR "Employment"[Mesh:NoExp] OR employment[tw] OR employability[tw] OR work resumption[tw] OR working age[tw] OR "job satisfaction"[Mesh] OR job satisfaction[tw] OR "sick leave"[Mesh] OR sick leave[tw] OR absenteeism[Mesh] OR absenteeism[tw] OR work retention[tw] OR job retention[tw] OR job status[tw] OR work status[tw] OR paid work[tw] OR vocational status[tw] OR occupational status[tw] OR work functioning[tw] OR job functioning[tw] OR work capacity[tw] OR employment capacity[tw] OR work participation[tw] OR stay at work[tw] OR presenteeism[tw] OR work outcomes[tw] OR work ability[tw]
#4	#1 AND #2 AND #3
Limits:	Publication date from 01/01/2007-02/2017; English language or Dutch language.

PsycINFO search strategy

Search	Query
#1 Population of interest	"chronicity (disorders)"/ or chronic illness/ or (chronic disease$ or chronic health or chronic condition$ or chronic illness).ab,id,ti. or (long-term sickness or long-term sick or chronic patients).ab,id,ti. or disorders/ or (disorder or disorders).ab,id,ti. or disabilities/ or (disability or disabilities).ab,id,ti. or injuries/ or (injury or injuries).ab,id,ti.
#2 Person-related factors	self evaluation/ or (self evaluation or subjective health complaints or self-appraisal or health concerns or perceived health or illness perceptions).ab,id,ti. or health attitudes/ or health attitudes.ab,id,ti. or client attitudes/ or (client attitudes or patient beliefs or patient perceptions or perceived severity or disability perceptions or pain beliefs or perceived disability or perceived illness or perceived impairment or beliefs about illness or illness beliefs or illness representations or illness cognitions or illness identity or psychological factors).ab,id,ti. or motivation/ or motivation.ab,id,ti. or employee motivation/ or (motivated or unmotivated or willingness to work or meaning of work).ab,id,ti. or "work (attitudes toward)"/ or work attitudes.ab,id,ti. or employee attitudes/ or (employee attitudes or work motives or work reasons or work drive).ab,id,ti. or (rehabilitation expectation$ or patient expectation$ or prognostic expectation$ or improvement expectation$ or recovery expectation$ or return to work expectation$ or RTW expectation$ or negative expectation$ or positive expectation$ or expected outcome or perceived curability or perceived work ability).ab,id,ti. or optimism/ or optimism.ab,id,ti. or pessimism/ or pessimism.ab,id,ti. or irrational beliefs/ or (irrational beliefs or irrational cognitions or irrational thoughts or irrational feelings or irrationality).ab,id,ti. or negativism/ or catastrophizing/ or (negativism or catastrophization or catastrophizing or cognitive insufficiency or negative perceptions or negative orientation or negative thoughts).ab,id,ti. or client participation/ or (client participation or patient participation or shared decision making or involvement in decision making or participation in decision making or informed decision-making or patient preference$).ab,id,ti. or "internal external locus of control"/ or (locus of control or internal-external control or control orientation or control beliefs or perceptions of control or illness controllability or perceived cause$ or external orientation or internal orientation or work-related$).ab,id,ti. or coping behavior/ or (coping or psychological adaption or fear-avoidance or adaptive response or avoidance behavior).ab,id,ti. or cognitive appraisal/ or cognitive appraisal.ab,id,ti. or self-concept/ or self-concept.ab,id,ti. or self-efficacy/ or self-efficacy.ab,id,ti. or self-confidence/ or self-confidence.ab,id,ti. or self-esteem/ or (self-esteem or perceived ability or self-image or perceived competence).ab,id,ti.
#3 Work participation outcome	reemployment/ or (return to work or (return to adj3 work) or back to work or RTW).ab,id,ti. or unemployment/ or unemployment.ab,id,ti. or employment status/ or (employment or work resumption or working age or paid work or work functioning or job functioning).ab,id,ti. or occupational status/ or (occupational status or job status or work status or vocational status or work participation or stay at work or presenteeism or work outcomes or work ability).ab,id,ti. or employability/ or (employability or work capacity or employment capacity).ab,id,ti. or job satisfaction/ or (job satisfaction or work retention or job retention).ab,id,ti. or employee absenteeism/ or (employee absenteeism or sick leave or absenteeism).ab,id,ti.
#4	#1 and #2 and #3
Limits:	Publication date from 01/01/2007-02/2017; English language or Dutch language.
