# Supplementary material for: Person-related factors associated with work participation in employees with health problems: a systematic review
Source: Int Arch Occup Environ Health. 2018 Apr 26;91(5):497–512. doi: 10.1007/s00420-018-1308-5 (PMC6002456; doi:10.1007/s00420-018-1308-5)
Supplement: Supplementary file 2 — Supplementary material 2 (PDF 540 KB) [file 420_2018_1308_MOESM2_ESM.pdf]

Person-related factors associated with work participation in employees with health problems: a systematic review

M. de Wit, H. Wind, C. T. J. Hulshof, M. H. W. Frings-Dresen

Department Coronel Institute of Occupational Health, Academic Medical Center, University of Amsterdam, Amsterdam Public Health research institute, Amsterdam, the Netherlands

Correspondence to: Mariska de Wit, Department Coronel Institute of Occupational Health, Academic Medical Center, University of Amsterdam, Amsterdam Public Health research institute, PO Box 22700, NL-1100 DE Amsterdam, The Netherlands; m.e.dewit@amc.uva.nl; +31(0)20-5665341

Journal: International Archives of Occupational and Environmental Health

Online Resource 2

Online Resource 2

Data extraction tables

Table 1

Association between person-related factor 'expectations regarding recover or RTW' and work participation

**First author, year, country (continent)**	**Study design**	**Population**<br>**N: Number of subjects**<br>**A: Age; mean age (SD), range**<br>**G: Gender**<br>**O: Occupation**<br>**H: Health status**	**Follow-up**	**Risk of bias**	**Results**
Audhoe et al. 2012<br>The Netherlands<br>(Europe)	Prospective cohort study	N: 932<br>A: 18-34: 232, 35-44: 260, 45-65: 418<br>G: 398 males, 514 females<br>O: Agency workers<br>H: Psychological complaints	10, 18, 27 months	Low	Positive RTW expectations at 10 months were associated with work participation at 18 months (*p* <. 01) and positive RTW expectations at 18 months were associated with work participation at 27 months (*p* < .01) in univariate analysis. Positive RTW expectations at 10 months were a prognostic factor for work participation at 18 months (OR: 1.7 (1.08-2.71), *p* = .02) in multiple logistic analysis. Positive RTW expectations at 18 months were a prognostic factor for work participation at 27 months (OR: 2.6 (1.12-5.86), *p* = .03) in multiple logistic analysis.
Besen et al. 2015<br>United States<br>(North-America)	Prospective cohort study	N: 241<br>A: 38 (11.4), 18-63<br>G: 130 males, 111 females<br>O: 76% blue collar<br>H: Low back pain	1 week, 3 months	Moderate	More favorable RTW expectations were negatively related to days of absence (*r* = -.19, *p* < .01) and negatively related to work status (*r* = -.42, *p* < .001) in univariate analysis (correlations). In multivariate analysis positive RTW expectations were associated with fewer days of absence (-.19, *p* < .05) and with work status (.35, *p* < .001).
Boot et al. 2008<br>The Netherlands<br>(Europe)	Cross-sectional study	Employed<br>N: 345<br>A: 44.2 (10.2)<br>G: 168 males, 177 females<br>O: -<br>H: Chronic physical disease<br>Fully work-disabled<br>N: 170<br>A: 52.4 (8.6)<br>G: 66 males, 104 females<br>O: -<br>H: Chronic physical disease	-	Low	The participants in the employed group had more positively oriented illness perceptions about how long their illness would last (*p* =. 027) and about the effect of their treatment (*p* < .001) compared to those who were fully work-disabled in univariate analysis. Those factors had no effect in multivariate logistic regression analysis.
Busch et al. 2007<br>Sweden<br>(Europe)	Prospective cohort study	Sick Absent<br>N: 130<br>A: <45: 64, >45: 66<br>G: 45 males, 85 females<br>O: -<br>H: Chronic musculoskeletal	3, 7, 11, 15 months	Low	Expectations of recovery were a significant predictor of long-term sickness absence in univariate analysis (*p* < .05). Having low expectations of recovery was a significant predictor of long-term sickness absence during follow-up (B = .88, OR: 2.41 (1.22-4.78), *p* = .01) in multivariate logistic regression analysis.

		pain<br>Work Capable<br>N: 103<br>A: <45: 58, >45: 45<br>G: 32 males, 71 females<br>O: -<br>H: Chronic musculoskeletal pain			
Carriere et al. 2015a<br>Canada<br>(North-America)	Prospective cohort study	N: 154<br>A: 36.4 (9.2), 20-60<br>G: 81 males, 73 females<br>O: Diverse<br>H: Whiplash associated disorders	1 year	Low	High RTW expectations were directly related to successful RTW ($\beta = 1.05$, $p < .001$) in multivariate analysis. Pain catastrophizing influenced RTW expectations ($p < .001$). Expectations partially mediated the relation between pain catastrophizing and RTW status in multiple regression analysis.
Carriere et al. 2015b<br>Canada<br>(North-America)	Prospective cohort study	N: 109<br>A: 36.08 (9.70)<br>G: 47 males, 62 females<br>O: Diverse<br>H: Work-related musculoskeletal disorders	1 year	Low	Lower recovery expectations were associated with lower probability of RTW ($\chi^2 = 36.75$, $p < .001$) in univariate Chi-square analysis. Recovery expectations contributed significantly to the prediction of RTW status at follow-up ($\chi^2 = 31.51$, OR .952 (.932–.972), $p < .001$) in multivariate logistic regression analysis. Low recovery expectations were associated with a low probability of RTW. Recovery expectations fully mediated the relation between depression and RTW status at follow-up in logistic regression analysis.
Carstens et al. 2014<br>United States<br>(North-America)	Prospective cohort study	N: 496<br>A: 37.0 (11.3), 18-65<br>G: 287 males, 209 females<br>O: -<br>H: Work-related acute back pain	3 months	Moderate	Four different recovery clusters were identified: having stable high expectations, having stable medium expectations, having decreasing expectations and having stable low expectations. The participants with stable low expectations for recovery had a higher risk for not returning to work within 3 months (OR: 3.38 (2.40-8.18), $p < .01$) as compared to participants with stable high expectations for recovery. The participants with decreasing expectations had the highest risk for not returning to work (OR: 5.29 (2.11-13.26), $p < .01$) in comparing to the other participants in multivariate logistic regression analysis. There was no difference between the participants in the stable high cluster and the participants in the stable medium cluster.
Coggon et al. 2013<br>Diverse<br>(Europe, South-North-America, South-America, Africa, Asia, Oceania)	Cross-sectional study	N: 12416<br>A: 20-29: 3058, 30-39: 3971, 40-49: 3451, 50-59: 1936<br>G: 4348 males, 8068 females<br>O: Diverse<br>H: Musculoskeletal illness	-	Moderate	Adverse beliefs about the prognosis of pain did not have a significant effect on prolonged sickness in the full model (PRR: 1.03 (.86-1.23)) in Poisson regression analysis.
Cowan et al. 2012<br>United States<br>(North-America)	Prospective cohort study	N: 66<br>A: 49.7 (11.3)<br>G: 17 males, 49 females<br>O: Desk-based, non-desk-based<br>H: Carpal tunnel release	2-4 months	Low	Less expected time until RTW ($p = .001$) was associated with earlier return to modified work in bivariate analysis.<br>In multivariate analysis less expected time until RTW ($R^2 = .36$) was significantly associated with earlier return to modified work. In bivariate analysis less expected time until RTW ($p < .001$) was significantly associated with earlier return to full work. Less expected time until RTW was also a significant predictor of earlier return to full work ($R^2 = .18$) in multivariate analysis.
Du Bois et al. 2009<br>Belgium<br>(Europe)	Prospective cohort study	N: 346<br>A: 41<br>G: 183 males, 163 females<br>O: 74% blue collar<br>H: Low back pain	6 months	Low	Expecting to RTW within 6 months was positively associated to RTW within 3 months (OR: 1.14 (1.04–1.25)) in multivariate analysis. The question ‘Do you expect to RTW within 6 months?’ was together with 4 other questions able to correctly classify the poor outcome (no RTW within 3 months) of 62% of the participants in multivariate logistic regression analysis.
Ekberg et al. 2015<br>Sweden<br>(Europe)	Prospective cohort study	RTW <3 months<br>N: 195<br>A: 44 (11), 18-65	1 year	Low	Participants who returned to work within 3 months had higher expectations of recovery from treatment than participants who returned to work between 3 and 12 months ($p = .035$) in univariate Chi-square analysis. Expectations of recovery from treatment were also associated with RTW in the 3-month sub-cohort, but not in the 3-12 month sub-cohort in multivariate Cox regression

		G: 38 males, 157 females<br>O: 77 white collar, 89 pink collar, 29 blue collar<br>H: Common mental disorders<br>RTW 3-12 months<br>N: 98<br>A: 44 (10), 18-65<br>G: 14 males, 84 females<br>O: 47 white collar, 34 pink collar, 16 blue collar<br>H: Common mental disorders			analysis when looking at the influence of personal resources. Positive expectations of recovery from treatment were associated with early RTW in the final model (HR: 1.50 (1.04-2.16)) in multivariate Cox regression analysis.<br>Expectations of RTW did not differ between the two groups in univariate analysis and were not associated with RTW in multivariate analysis.
Gross and Battié 2010<br>Canada<br>(North-America)	Prospective cohort study	Specific pathology:<br>N: 234<br>A: 38.1 (11.6)<br>G: 199 males, 35 females<br>O: -<br>H: Specific pathology<br>Back pain:<br>N: 298<br>A: 38.1 (10.6)<br>G: 203 males, 95 females<br>O: -<br>H: Back pain<br>Non-back strain/sprain/pain<br>N: 461<br>A: 40.5 (10.4)<br>G: 290 males, 171 females<br>O: -<br>H: Non-back strain/sprain/pain<br>Other pain<br>N: 47<br>A: 40.7 (9.1)<br>G: 28 males, 19 females<br>O: -<br>H: Other pain	1 year	Moderate	Positive work related recovery expectations were significantly associated with more days to suspension of time loss benefits in participants with back pain in univariate ($p < .05$) and in multivariate analysis (OR: .83 (.73-.96), $p < .05$), but not in other diagnostic groups in multivariate analysis. Participants with back pain and with positive expectations recover more rapidly, than participants with back pain with neutral or negative expectations.<br>Expectations did influence the time to claim closure in univariate analysis for participants with back pain ($p < .05$), but did not influence time to claim closure in other diagnostic groups or in multivariate analysis.<br>When taking the non-chronic and chronic back pain groups apart, recovery expectations were associated with time to benefit suspension in univariate ($p < .05$) and in multivariate analysis (OR: .76 (.62-.92), $p < .05$) in the non-chronic back pain group, but not in the chronic back pain group. Recovery expectations were associated with time to claim closure in univariate ($p < .05$) and in multivariate analysis (OR: .80 (.66-.98), $p < .05$) in the non-chronic back pain group, but not in the chronic back pain group.
Hou et al. 2012<br>Taiwan<br>(Asia)	Prospective cohort study	N: 804<br>A: 41.8 (15.2), 20-65<br>G: 574 males, 230 females<br>O: White-collar, blue-collar, part-time job<br>H: Traumatic limb injury	1, 3, 6, 12, 18, 24 months	Low	The participants who expected that the likelihood for RTW within one month was moderate (OR: .29, (.17-.51)) or high (OR: .20 (.09-.47)) were more likely to be in the fast RTW group than in the slow RTW group, in comparing to participants who expected that there was no chance for RTW within one month. The participants who expected that the likelihood for RTW within one month was moderate (OR: .52 (.34-.80)) or high (OR: .34 (.17-.69)) were more likely to be in the average RTW group than the slow RTW group in multinomial logistic regression analysis. There were no significant differences in expectations for RTW between participants in the average RTW and the fast RTW group.
Hou et al. 2008<br>Taiwan<br>(Asia)	Prospective cohort study	N: 154<br>A: 36.9 (10.9), 18-65<br>G: 115 males, 39 females<br>O: White-collar and blue-collar<br>H: Orthopaedic injury	1, 3, 6 months	Low	For non-workers' compensation group expecting that the likelihood for RTW within one month is high, was associated with a greater chance of RTW in univariate and multivariate analysis (OR: 4.88 (2.31-10.30)). For workers' compensation group high expectations (OR: 2.87 (1.11-13.15)) and very high expectations (OR: 11.14 (2.40-51.59)) for RTW within one month were associated with a greater chance of RTW in univariate and in multivariate Cox regression analysis.
Iakova et al. 2012	Prospective	N: 411	2 years	Moderate	Expected injury outcome (recovering vs. no recovery or worsening) was positively associated with RTW in a univariate regression

Switzerland<br>(Europe)	cohort study	A: 43.3 (10.3), <60<br>G: 336 males,75 females<br>O: -<br>H: Orthopedic trauma			(*p* < .001), but was not a significant predictor of RTW in the full model in multivariate analysis.
Jensen et al. 2013<br>Denmark<br>(Europe)	Prospective cohort study	N: 325<br>A: 41.7 (10.4), 18-60<br>G: 159 males, 166 females<br>O: -<br>H: Low back pain	1 year	Low	Low expectations for RTW (*p* < .001) predicted unsuccessful RTW after one year in univariate analysis. Low RTW expectations were in combination with five other variables also negatively associated with RTW in multivariate logistic regression analysis (*p* < .001).
Johansson et al. 2010<br>Sweden<br>(Europe)	Prospective cohort study	N: 59<br>A: 40 (8), 18-60<br>G: 35 males, 24 females<br>O:-<br>H: Lumbar disc herniation	1 year	Low	Having low expectations to RTW within 3 months was a predictor of sick leave 12 months after surgery (OR: 19.5 (2.1-179.2), *p* = .009) in multivariate logistic regression analysis.
Lindell et al. 2010<br>Sweden<br>(Europe)	Prospective cohort study	Rehabilitation group<br>N: 63<br>A: 42.2 (2.4), <60<br>G: 30 males, 33 females<br>O: 87% blue collar<br>H: non-acute non-specific spinal pain<br>Primary-care group<br>N: 62<br>A: 43.0 (2.6), <60<br>G: 27 males, 35 females<br>O: 87% blue collar<br>H: non-acute non-specific spinal pain	6, 12, 18, 24 months	Low	The participants' own belief in RTW was a significant predictor of RTW at 6 months (*p* = .03), 12 months (*p* = .002), 18 months (*p* = .005), and 24 months (*p* = .008) in univariate analysis.<br>The participants' own belief in RTW was a predictor of RTW at 6 months (OR: 4.1 (1.1-15.7), *p* = .02) and 12 months (OR: 5.2 (1.5-17.5), *p* = .009), but not at 18 months and 24 months in multivariate analysis.
Magnussen et al. 2007b<br>Norway<br>(Europe)	Randomized controlled trial	Intervention<br>N: 45<br>A: 49.1 (6.4), 36-56<br>G: 19 males, 26 females<br>O: -<br>H: Back pain<br>Control<br>N: 44<br>A: 49 (4.5), 36-56<br>G: 14 males, 30 females<br>O: -<br>H: Back pain	1 year	Moderate	Belief in RTW was associated with having entered in a RTW process (being on educational course or being in work training) in univariate analysis (*p* = .003). The participants who at baseline believed to RTW (OR: 10.5 (2.4-44.5), *p* < .05) were more likely to have entered a RTW process when adjusted for age and gender in multivariate logistic regression analysis.
Murgatroyd et al. 2016<br>Australia<br>(Oceania)	Prospective cohort study	N: 334<br>A: 36 (13.9), >18<br>G: 267 males, 67 females<br>O: Diverse<br>H: Upper and/or lower extremity fractures	6, 12, 24 months	Moderate	Recovery expectations for work (*p* = .08) and recovery expectations for usual activities (*p* < .001) were associated with RTW in univariate analysis. Expecting that one could perform usual activities in less than 90 days was associated with a shorter time to RTW (HR: 2.10 (1.49-2.95), *p* < .001) in comparing to expecting that one could not perform usual activities within 90 days in the Cox model in multivariate analysis. However, recovery expectations for work were not associated with RTW in multivariate Cox regression analysis.

Nieuwenhuijsen et al. 2013<br>The Netherlands<br>(Europe)	Retrospective cohort study	N: 179<br>A: 45 (10)<br>G: 51 males, 128 females<br>O: -<br>H: Common mental disorders	3, 6, 9, 12 months	Moderate	Participants with positive RTW expectations did not differ in median time till RTW in comparing to workers with negative RTW expectations in a univariate log-rank test. Positive RTW expectations were not significantly associated with a shorter median time to RTW.
Opsahl et al. 2016<br>Norway<br>(Europe)	Prospective cohort study	N: 574<br>A: 44.3 (9.7), 20-60<br>G: 285 males, 289 females<br>O: -<br>H: Low back pain	1 year	Low	Having high expectations of RTW instead of low or moderate expectations predicted RTW at 12 months for men ($p < .05$) and women ($p < .05$) in bivariate analysis and for men (OR: 4.17 (1.90-9.17)) and women (OR: 3.36 (1.58-7.14)) in the full model in multivariate analysis. Having uncertain expectations were no better than having low or moderate expectations in bivariate logistic regression analysis.
Reme et al. 2009<br>Norway<br>(Europe)	Prospective cohort study	N: 246<br>A: 41.1 (10.7), <60<br>G: 120 males, 126 females<br>O: -<br>H: Low back pain	3, 12, 24 months	Low	Negative RTW expectations predicted non RTW in univariate analysis ($p < .01$). In adjusted regression models negative RTW expectations predicted non RTW at 3 months (OR: 4.2 (1.7-10.0), $p = .001$), but did not predict non RTW at 12 or 24 months.
Richter et al. 2011<br>The Netherlands<br>(Europe)	Prospective cohort study	N: 276<br>A: 45 (7)<br>G: 256 males, 20 females<br>O: Self-employed<br>H: Musculoskeletal symptoms	1 year	Low	Participants who estimated their RTW to be within one month had shorter claim duration than participants who estimated RTW in more than a month or never (OR: .24 (.15-.38), $p < .001$) or who had no idea of when they would RTW (OR: .23 (.15-.34), $p < .001$) in univariate and in multivariate Cox regression analysis.
Rönnberg et al. 2007<br>Sweden<br>(Europe)	Prospective cohort study	N: 148<br>A: 40, 18-66<br>G: 80 males, 68 females<br>O: -<br>H: Lumbar disc hernia	2 years	Moderate	Participants who expected to return (76%) and not return (24%) to their present or similar work, returned in 78% and 26%, respectively ($p = .021$) in univariate analysis.
Sampere et al. 2012<br>Spain<br>(Europe)	Prospective cohort study	N: 663<br>A: 39.8 (11.4)<br>G: 364 males, 299 females<br>O: -<br>H: Musculoskeletal disorder, mental disorders or other physical conditions	2 years	Low	Expectations of returning to the same job were associated with time to RTW in bivariate analysis ($p < .005$). Expecting that the time required to RTW was1-3 months (HR: .50 (.39-.63)) or was more than 3 months (HR: .36 (.25-.52)) was predictive for more time to RTW in comparing to expecting that the time required to RTW was less than 1 months. Lack of expectations of returning to the same job (HR: .13 (.06-.31)) and not knowing when to return (HR: .46 (.37-.57)) were also associated with a longer time to RTW in the full model among the total study population in multivariate analysis. RTW expectations were related to time to RTW across different health conditions and across different genders in multivariate analysis.
Sluiter and Frings-Dresen 2008<br>The Netherlands<br>(Europe)	Cross-sectional study	Employed<br>N: 745<br>A: 40.0 (8.3)<br>G: 283 males,462 females<br>O: -<br>H: Repetitive strain injury<br>Sick-listed<br>N: 376<br>A: 42.3 (9.4)<br>G: 83 males, 293 females<br>O: -<br>H: Repetitive strain injury	-	Low	Participants with repetitive strain injury who were sick listed differed from the working group in treatment control ($p < .001$). Sick-listed participants scored lower on treatment control than participants in the working group which indicated that they had less positive expectations about the effect of their treatment in multivariate ANOVA analysis. However they did not differ in their perceptions about how long their illness would last.
Spector et al. 2012<br>United States<br>(North-America)	Prospective cohort study	N: 670<br>A: 44.9 (9.6), >18<br>G: 255 males, 415 females	2 years	Low	Low or uncertain recovery expectations were significantly associated with long-term disability (missing work days and retrieving compensation) in univariate ($p < .05$) and in multivariate analysis (OR: 2.51 (1.39-4.54), $p = .002$).

		O: Diverse<br>H: Carpal tunnel syndrome			
Truchon et al. 2012<br>Canada<br>(North-America)	Prospective cohort study	N: 535<br>A: 42 (10), 18-60<br>G: 317 males, 218 females<br>O: Diverse<br>H: Low back pain	6, 12 months	High	Expectations of time to RTW ($\beta = -.25$, $p = .00$) as measured with the new developed absenteeism screening questionnaire were predictive for long-term absence from work in multivariate Cox regression analysis.
Turner et al. 2008<br>United States<br>(North-America)	Prospective cohort study	N: 1885<br>A: 39.4 (11.2)<br>G: 1282 males, 603 females<br>O: Diverse<br>H: Back injury	1 year	Low	Having low or high instead of very high recovery expectations was associated with work disability ($p < .001$) in univariate analysis. In a multi-domain model none of the psychological factors, including recovery expectations, contributed independently to the prediction of 1-year work disability (wage replacement compensation for total disability 12 months after claim submission). This was because the score on the Roland-Morris disability questionnaire was substantially correlated with all the psychological factors.
Vuistiner et al. 2015<br>Switzerland<br>(Europe)	Prospective cohort study	N: 1090<br>A: 42.9 (11.3), <62<br>G: 890 males, 200 females<br>O: -<br>H: Orthopaedic trauma	4 years	Low	The probability of being declared fit for work was higher for those expecting a positive evolution in univariate analysis and in multivariate Cox regression analysis (HR: 1.50 (1.32-1.70)).
Wåhlin et al. 2012<br>Sweden<br>(Europe)	Prospective cohort study	N: 699<br>A: 48 (10.7), 18-65<br>G: 201 males, 498 females<br>O: -<br>H: Musculoskeletal or mental disorders	3 months	Low	For participants with mental disorders there was no association between RTW expectations and RTW. For participants with musculoskeletal disorders high RTW expectations were significantly associated with RTW within 3 months (OR: 2.41 (1.22-4.78)) in multiple logistic regression analysis.

* RTW: Return to work

Table 2

Association between person-related factor 'optimism' and work participation

**First author, year, country (continent)**	**Study design**	**Population**<br>**N: Number of subjects**<br>**A: Age; mean age (SD), range**<br>**G: Gender**<br>**O: Occupation**<br>**H: Health status**	**Follow-up**	**Risk of bias**	**Results**
De Vries et al. 2011<br>The Netherlands<br>(Europe)	Qualitative study	N: 21<br>A: 49 (6.9), 10-60<br>G: 9 males, 12 females<br>O: Diverse<br>H: Chronic nonspecific musculoskeletal pain	-	Low	A positive outlook was according to participants with chronic nonspecific musculoskeletal pain an important factor for staying at work.
Ellingsen and Aas 2009<br>Norway<br>(Europe)	Qualitative study	N: 4<br>A: 38-52<br>G: 2 males, 2 females<br>O: -<br>H: Acquired brain injury	-	Moderate	A belief that things would work out (optimism) was considered as a very important facilitating factor for work participation according to participants. Pessimism was a inhibiting factor for work participation.
Hystad and Bye 2012<br>Norway<br>(Europe)	Prospective cohort study	N: 1190<br>A: 41.67 (10.87)<br>G: 922 males, 268 females<br>O: Diverse<br>H: Musculoskeletal symptoms	2 years	Low	Pessimism was related to more sickness absence in 2 years for women ($R^2 = .12$, $p < .01$) after controlling for age, physical work environment and proposed mediators, but not for men ($R^2 = .04$, $p = .057$) in multivariate analysis. However, the coefficient did not significantly differ between men and women. There was no effect for optimism. Pessimism was a more salient predictor of physical health and functioning than optimism in a multiple mediation model.
Lundqvist and Samuelsson 2012<br>Sweden<br>(Europe)	Qualitative study	N: 14<br>A: 51 (8.1), 37-63<br>G: 8 males, 6 females<br>O: -<br>H: Brain injury	-	Moderate	According to participants it was important to have a positive attitude for progressing in rehabilitation and RTW.
Lydell et al. 2011<br>Sweden<br>(Europe)	Prospective cohort study	N: 320<br>A: 42<br>G: 130 males, 190 females<br>O: Diverse<br>H: Musculoskeletal disorders	1, 5, 10 years	Moderate	There were more participants in the motivation and optimism group who had returned to work 1 year after baseline as compared to the hindrance and hesitation group. However, there were no significant differences between the groups after 5 and 10 years in univariate Chi-square analysis.
Øyeflaten et al. 2008<br>Norway<br>(Europe)	Prospective cohort study	N: 135<br>A: 45 (8.4), 24-61<br>G: 48 males, 87 females<br>O: Diverse<br>H: Musculoskeletal complaints	3, 12 months	Low	Hopelessness (having negative expectancies about oneself and the future) had no significant effect on RTW at 3 and 12 months in univariate or multivariate logistic regression analysis.

* RTW: Return to work

Table 3

Association between person-related factor 'self-efficacy' and work participation

**First author, year, country (continent)**	**Study design**	**Population**<br>**N: Number of subjects**<br>**A: Age; mean age (SD), range**<br>**G: Gender**<br>**O: Occupation**<br>**H: Health status**	**Follow-up**	**Risk of bias**	**Results**
Besen et al. 2015<br>United States<br>(North-America)	Prospective cohort study	N: 241<br>A: 38 (11.4), 18-63<br>G: 130 males, 111 females<br>O: 76% blue collar<br>H: Low back pain	1 week, 3 months	Moderate	Greater RTW confidence (confidence in ability to RTW) was negatively related to days of absence (r = -.34, *p* < .001) and positively related to work status (r = .32, *p* < .001) in univariate analysis (correlations). In multivariate analysis greater RTW confidence (r = -.10, *p* < .05) was associated with fewer days of absence. Greater RTW confidence (r = .09, *p* < .05) was also directly associated with work status.
Brouwer et al. 2015<br>Canada<br>(North-America)	Prospective cohort study	N: 632<br>A: 15-29: 93, 30-39: 137, 40-49: 228, >49: 173<br>G: 350 males, 282 females<br>O: -<br>H: Musculoskeletal disorders	1, 6, 12 months	Low	High levels of pain RTW self-efficacy (ability to cope with pain) as measured with the new return-to-work self-efficacy scale 1 month after injury was positively related to 6-month RTW status in univariate (*p* < .001) and in multivariate analysis (β = .18, OR: 1.20 (1.06-1.37), *p* = .005). Overall RTW self-efficacy was positively associated with 6 month RTW status in univariate (*p* = .004), but not in multivariate analysis. Other self-efficacy scales (supervisor RTW self-efficacy and co-worker RTW self-efficacy) were not significant in predicting 6-months RTW status in univariate or multivariate analysis.<br>Overall RTW self-efficacy (*p* = .015) and pain RTW self-efficacy (*p* = .029) predicted 12 month RTW status in univariate analysis, but not in multivariate analysis. Supervisor RTW self-efficacy and co-worker self-efficacy did not predict 12 month RTW status in univariate or multivariate logistic regression analysis.<br>Improvements in overall RTW self-efficacy (β = .66, OR: 1.92 (1.04-3.57), *p* = .038) and coworker RTW self-efficacy (ability to obtain help from coworkers) (β = .89, OR: 2.43 (1.18-5.00), *p* = .016) predicted 12-month RTW status, but only in multivariate logistic regression analysis. Other improvements of forms of RTW self-efficacy (pain RTW self-efficacy and supervisor RTW self-efficacy) were not significant in predicting RTW status in univariate or multivariate analysis.
Brouwer et al. 2009<br>The Netherlands<br>(Europe)	Prospective cohort study	N: 926<br>A: 45.8 (9.5), 18-63<br>G: 466 males, 460 females<br>O: -<br>H: Musculoskeletal symptoms, other physical symptoms, mental symptoms	10 months	Low	Willingness to expend effort in completing a behavior (*p* = .00) and willingness to initiate behavior (*p* = .01) (two subscales of self-efficacy) were associated with less time till RTW in univariate analysis. The subscale persistence in the face of adversity was not associated with time till RTW in univariate analysis. Willingness to expend effort in completing the behavior was associated with shorter time till RTW (HR: 1.42 (1.17-1.74), *p* = .00) in multivariate analysis. Other subscales (willingness to initiate behavior and persistence in the face of adversity) were not significant in multivariate analysis.
Brouwer et al. 2010<br>The Netherlands<br>(Europe)	Prospective cohort study	Musculoskeletal conditions<br>N: 352<br>A: 45.4 (9.4)<br>G: 201 males, 151 females<br>O: -<br>H: Musculoskeletal conditions<br>Other physical health conditions<br>N: 265<br>A: 47.7 (9.5)<br>G: 148 males, 117 females	10 months	Low	High willingness to expend effort in completing a behavior was a significant predictor of shorter time to RTW for all three subgroups (musculoskeletal, other physical and mental health subgroup) in univariate analysis (*p* < .05). Willingness to initiate behavior was only associated with shorter time till RTW for the musculoskeletal subgroup in univariate analysis (*p* < .01), but not for the other physical subgroup and the mental health subgroup. The subscale persistence in the face of adversity was not associated with time to RTW in all three groups.<br>Willingness to expend effort in completing a behavior (self-efficacy) was associated with time to RTW after 10 months (*p* < .05) in all three groups participants (musculoskeletal subgroup: HR 1.49 (1.12-1.99), *p* < .01, other physical subgroup: HR: 1.53 (1.07-2.18), *p* = .02, mental health subgroup: HR: 1.60 (1.07-2.40), *p* = .02) in the multivariate model. Other subscales of self-efficacy (willingness to initiate behavior and persistence in the face of adversity) were not significantly associated with time to RTW in multivariate analysis.

		O: -<br>H: Other physical health conditions<br>Mental health conditions<br>N: 245<br>A: 44.2 (9.4)<br>G: 86 males, 159 females<br>O: -<br>H: Mental health conditions			
D'Amato and Zijlstra 2010<br>Austria, Ireland, Finland, the Netherlands, the United Kingdom (Europe)	Prospective cohort study	N: 1460<br>A: <36: 210 , 36-45: 337, 46-54: 515, >55: 396<br>G: 711 males, 749 females<br>O: Diverse<br>H: Mental or physical health problems	6 months	Moderate	Self-efficacy was not significant in predicting RTW in multivariate logistic regression analysis.
De Vries et al. 2011<br>The Netherlands (Europe)	Qualitative study	N: 21<br>A: 49 (6.9), 10-60<br>G: 9 males, 12 females<br>O: Diverse<br>H: Chronic nonspecific musculoskeletal pain	-	Low	Being self-confident was according to participants with chronic nonspecific musculoskeletal pain an important factor for staying at work.
De Vries et al. 2012b<br>The Netherlands (Europe)	Cross-sectional study	Staying at work<br>N: 119<br>A: 51, 20-60<br>G: 48 males, 71 females<br>O: -<br>H: Chronic nonspecific musculoskeletal pain<br>Sick leave and referred for rehabilitation<br>N: 122<br>A: 39, 20-60<br>G: 56 males, 66 females<br>O: -<br>H: Chronic nonspecific musculoskeletal pain	-	Low	Pain self-efficacy (higher in staying at work group) was associated with work status in univariate analysis (p = .001). Participants in the staying at work group reported significantly higher pain self-efficacy beliefs compared to sick-listed participants in the sick-leave group (B = .09, OR: 1.09 (1.05-1.14), p = .001) in multivariate logistic regression analysis. However, self-efficacy was not one of the factors which best discriminated participants within the sick leave group and the staying at work group in backwards stepwise logistic regression analysis.
Dionne et al. 2007<br>Canada (North-America)	Prospective cohort study	N: 1007<br>A: 38.7 (10.6), 18-64<br>G: 589 males, 418 females<br>O: -<br>H: Back pain	6 weeks, 12 weeks, 1 year, 2 years	Low	Self-efficacy was associated with less failure after attempt(s) to RTW for women (OR: .69 (.51–.91), $p < .05$) and men (OR: .21 (.07–.68), $p < .05$) in multivariate analysis. However, self-efficacy was not associated with partial success or failure to RTW.
Dunn et al. 2010<br>United States (North-America)	Qualitative study	N: 23<br>A: 27-59<br>G: 11 males, 12 females<br>O: Diverse<br>H: Serious mental illness	-	Moderate	Seven facilitators of vocational recovery were received from the interviews. Having the confidence to work was important in helping participants with serious mental illness RTW and maintain their employment.

Ekberg et al. 2015<br>Sweden<br>(Europe)	Prospective cohort study	RTW <3 months<br>N: 195<br>A: 44 (11), 18-65<br>G: 38 males, 157 females<br>O: 77 white collar, 89 pink collar, 29 blue collar<br>H: Common mental disorders<br>RTW 3-12 months<br>N: 98<br>A: 44 (10), 18-65<br>G: 14 males, 84 females<br>O: 47 white collar, 34 pink collar, 16 blue collar<br>H: Common mental disorders	1 year	Low	Self-efficacy did not differ between participants who went back to work within 3 month and participants who went back to work between 3 and 12 months in univariate Chi-square analysis. Self-efficacy was related to RTW in the 3-12 months cohort, but not in sub-cohort for participants who returned to work within 3 months in multivariate Cox regression analysis.<br>Significant variables from multiple Cox regression within each block of predictors (health, function and work ability, personal resources and work conditions) were included in the full model. Self-efficacy was not associated with early RTW in the final model in multivariate Cox regression analysis.
Hartke et al. 2011<br>United States<br>(North-America)	Qualitative study	N: 12<br>A: 51, 31-67<br>G: 8 males, 4 females<br>O: Diverse<br>H: Stroke	-	Moderate	Struggling with regaining self-confidence was a barrier that stroke survivors encounter in their efforts to RTW.
Healey et al. 2011<br>United Kingdom<br>(Europe)	Cross-sectional study	N: 612<br>A: 50.8 (12.2)<br>G: 438 males, 174 females<br>O: -<br>H: Ankylosing spondylitis	-	Low	Self-efficacy was negatively associated with unemployment and absenteeism (number of sick leave days) in univariate analysis ($p < .05$), but was not associated with employment status and absenteeism in multivariate logistic regression analysis.
Huijs et al. 2012<br>The Netherlands<br>(Europe)	Prospective cohort study	N: 682<br>A: 46.6 (9.25)<br>G: 284 males, 389 females<br>O: -<br>H: Physical problems, mental problems or a combination of physical and mental problems	2 years	Low	Less RTW self-efficacy was associated with a longer duration until full RTW for participants with physical problems, and physical and mental problems in univariate analysis ($p < .01$), but not for participants with only mental problems. Self-efficacy was associated with duration until full RTW for participants with sickness absence due to physical complaints (HR: 1.38 (1.15-1.64), $p < .01$) and for participants with physical and mental problems (HR: 1.24 (1.01-1.51), $p = .04$), but not for participants with only mental problems in multivariate analysis.
Huijs et al. 2017<br>The Netherlands<br>(Europe)	Prospective cohort study	N: 883<br>A: 46.4 (9.2)<br>G: 386 males, 497 females<br>O: -<br>H: Long-term sick-listed employees with and without depressive symptoms	1 year, 2 years	Moderate	RTW self-efficacy predicted duration until RTW within 1 year and within 2 years in univariate analysis ($p < .01$). A higher level of RTW self-efficacy predicted a shorter duration until full RTW within 1year (HR: 1.19 (1.06-1.33), $p < .01$) or 2 years (HR: 1.20 (1.06-1.35), $p < .01$) after the start of sick leave for participants with depressive symptoms in multivariate analysis.
Lagerveld et al. 2016<br>The Netherlands<br>(Europe)	Prospective cohort study	N: 168<br>A: 40.7 (9.9), >50: 32<br>G: 67 males, 101 females<br>O: -<br>H: Common mental disorders	1, 3, 6, 9 months	Moderate	Higher baseline self-efficacy (HR: 3.16 (2.04-4.87), $p < .01$) and stronger self-efficacy increase (HR: 1.91 (1.46-2.53), $p < .01$) were significant predictors of a faster RTW within 9 months in multivariate Cox regression analysis.
Lundqvist and Samuelsson 2012<br>Sweden<br>(Europe)	Qualitative study	N: 14<br>A: 51 (8.1), 37-63<br>G: 8 males, 6 females<br>O: -	-	Moderate	According to participants it was important to have self-confidence for progressing in rehabilitation and RTW.

		H: Brain injury			
Magnussen et al. 2007a<br>Norway<br>(Europe)	Qualitative study	N: 17<br>A: 38-56<br>G: 5 males, 12 females<br>O: -<br>H: Back pain	-	Moderate	Low self-esteem was for many participants a main barrier for RTW.
Mangels et al. 2011<br>Germany<br>(Europe)	Prospective cohort study	Short-term sick leave<br>N: 161<br>A: 48.6 (13.7)<br>G: 39 males, 122 females<br>O: -<br>H: Musculoskeletal disease<br>Long-term sick leave<br>N: 53<br>A: 50.3 (6.5)<br>G: 12 males, 41 females<br>O: -<br>H: Musculoskeletal disease	1 year	Moderate	Pretreatment self-efficacy did not contribute to the prediction of sick leave at follow-up in multivariate analysis. Participants with low scores on self-efficacy at follow-up were more likely to be on long-term sick leave at follow-up than patients with higher scores on self-efficacy ($\beta = -.36$, $p < .001$) in hierarchical regression analysis.
Martins 2015<br>Portugal<br>(Europe)	Cross-sectional study	N: 149<br>A: 40.61 (11.13), 19-64<br>G: 109 males, 40 females<br>O: -<br>H: Chronic disease or injury, using a wheelchair	-	Moderate	Participants who are employed scored higher on self-efficacy than retired or unemployed participants ($t = 6.26$, $p < .001$) in a t-test.
Murphy et al. 2011<br>Australia<br>(Oceania)	Prospective cohort study	N: 72<br>A: 35.3 (14.4)<br>G: 58 males, 14 females<br>O: -<br>H: Spinal cord injury	2 years	Moderate	Self-efficacy was not a predictor of employment status in univariate analysis or multivariate discriminate function analysis.
Nieuwenhuijsen et al. 2013<br>The Netherlands<br>(Europe)	Retrospective cohort study	N: 179<br>A: 45 (10)<br>G: 51 males, 128 females<br>O: -<br>H: Common mental disorders	3, 6, 9, 12 months	Moderate	Positive RTW self-efficacy was associated with a shorter time until RTW ($\chi^2 = 17.8$, $p < .000$) in a Kaplan-Meier curve in survival analysis.
O'Sullivan et al. 2012<br>United states<br>(North-America)	Cross-sectional study	N: 56<br>A: 40.7 (13.2), 19-69<br>G: 23 males, 32 females<br>O: -<br>H: Physical disability, psychiatric disability, learning disability	-	Low	Collective work behavior self-efficacy was associated with length of prior employment. Participants who reported higher levels of work behavior self-efficacy reported longer periods of employment ($r = .36$, $p < .01$) in univariate analysis. However, self-efficacy was not an independent predictor of length of prior employment when adding personality factors in hierarchical regression analysis.
Øyeflaten et al. 2008<br>Norway<br>(Europe)	Prospective cohort study	N: 135<br>A: 45 (8.4), 24-61<br>G: 48 males, 87 females<br>O: Diverse<br>H: Musculoskeletal complaints	3, 12 months	Low	Self-efficacy had no significant effect on RTW at 3 and 12 months in univariate or multivariate logistic regression analysis.
Richard et al. 2011	Prospective	N: 1007	2 years	Low	Participants with high RTW self-efficacy were less likely to be found in the failure after attempt to RTW group (OR: .28 (.14-.57),

Canada<br>(North America)	cohort study	A: 38.7 (10.6), 18-64<br>G: 589 males, 418 females<br>O: -<br>H: Back pain			*p* < .05) or in the failure group (OR: .19 (.07-.48), *p* < .05) than participants with low self-efficacy in bivariate and multivariate analysis. Participants with a moderate level of RTW self-efficacy were less likely to be found in the failure after attempt (OR: .82 (.47-1.40), *p* < .05) or in the failure group at 2 years (OR: .51 (.26-1.00), *p* < .05) in univariate and multivariate analysis. There was no effect of self-efficacy and the chance of being in the partial success group.
Roesler et al. 2013<br>Australia<br>(Oceania)	Prospective cohort study	N: 192<br>A: 35.1, 18-63<br>G: 163 males, 29 females<br>O: Blue collar, white collar<br>H: Hand injury	4, 12 weeks	Low	Self-efficacy at 7-10 days was a negative predictor of failing to RTW before 12 weeks (B = -.21, Exp(B) = .81, *p* = .014), but self-efficacy at 28 days was not a predictor of RTW before 12 weeks in multivariate logistic regression analysis.
Sampere et al. 2012<br>Spain<br>(Europe)	Prospective cohort study	N: 663<br>A: 39.8 (11.4)<br>G: 364 males, 299 females<br>O: -<br>H: Musculoskeletal disorder, mental disorders or other physical conditions	2 years	Low	General self-efficacy was not related to time to RTW in bivariate analysis and not in multivariate analysis.
Sarda et al. 2009<br>Australia<br>(Oceania)<br>Brazil<br>(South-America)	Cross-sectional study	Australian<br>N: 207<br>A: 44, 18-65<br>G: -<br>O: -<br>H: Chronic pain<br>Brazil<br>N: 222<br>A: 45, 18-65<br>G: -<br>O: -<br>H: Chronic pain	-	Low	In the Brazilian sample low self-efficacy was associated with a more chance of being unemployed (OR: 2.52 (1.06-6.00), *p* = .04) in multivariate analysis. Self-efficacy was not associated with chance of being unemployed in the Australian sample in multivariate logistic regression analysis.
Shaw et al. 2011<br>United States<br>(North-America)	Prospective cohort study	N: 399<br>A: 36.5 (11.2), 18-63<br>G: 236 males, 163 females<br>O: Mostly blue-collar<br>H: Acute low-back pain	3 months	Moderate	Participants with medium (OR: 3.40 (1.58-7.33), *p* < .01) or high (OR: 4.93 (2.23-10.91), *p* < .01) RTW self-efficacy at visit 1 at the occupational health clinic were more likely to RTW within 7 days in univariate (*p* < .01) and multivariate analysis.<br>Self-efficacy at visit 1 was not a predictor of RTW within 3 months in univariate or multivariate analysis. High self-efficacy at visit 2 (4-10 days after first visit at the clinic) was a significant predictor of RTW within 3 months in univariate (*p* < .05) and multivariate analysis (OR: 3.72 (1.51-9.13), *p* < .01).
Strauser et al. 2010<br>United States<br>(North-America)	Cross-sectional study	N: 84<br>A: 40.7 (13.2), 19-69<br>G: 35 males, 48 females<br>O: -<br>H: Physical disabilities, psychiatric disabilities, learning disabilities	-	Low	Level of contextual work behavior self-efficacy was not different between employed and unemployed participants. Contextual work behavior self-efficacy did not contribute to predicting the longest period of prior employment next to work personality in hierarchical multiple regression analysis.
Stulemeijer et al. 2008<br>The Netherlands<br>(Europe)	Prospective cohort study	N: 201<br>A: 35.6 (12.3), 18-60<br>G: 123 males, 78 females<br>O: -<br>H: Mild traumatic brain injury	6 months	Low	Self-efficacy did not predict RTW in univariate analysis.
Tamminga et al.	Qualitative	N: 12	-	Low	Being confident was a RTW facilitator according to participants with breast cancer.

2012<br>The Netherlands<br>(Europe)	study	A: 42 (7), 18-65<br>G: 0 males, 12 females<br>O: -<br>H: Breast cancer			
Volker et al. 2015<br>The Netherlands<br>(Europe)	Prospective cohort study	N: 493<br>A: >44: 308 of 487<br>G: 220 males, 237 females<br>O: -<br>H: Long-term sick-listed	2 years	Low	Participants with high RTW self-efficacy had a shorter time to RTW compared to participants with low RTW self-efficacy (HR: 2.02 (1.50-2.73), $p < .01$) in bivariate Cox regression analysis. Higher RTW self-efficacy was not significantly associated with shorter duration until RTW (HR: 1.60 (1.12-2.28), $p = .010$) in multivariate analysis.
Waghorn et al. 2007<br>Australia<br>(Oceania)	Prospective cohort study	N: 104<br>A: 19-24: 12, 25-34: 37, 35-44: 28, 45-56: 27<br>G: 75 males, 29 females<br>O: -<br>H: Schizophrenia or schizoaffective disorders	6, 12 months	Moderate	Total work self-efficacy was associated with current employment in univariate analysis ($p < .001$) and in a multivariate mixed regression model (F = 5.84, $p = .02$). General work skills self-efficacy ($p < .001$), job securing skills self-efficacy ($p < .05$) and career planning skills self-efficacy ($p < .01$) were also associated with current employment status in univariate analysis. Work-related social skills self-efficacy was not related to current employment in univariate analysis.
Wåhlin et al. 2012<br>Sweden<br>(Europe)	Prospective cohort study	N: 699<br>A: 48 (10.7), 18-65<br>G: 201 males, 498 females<br>O: -<br>H: Musculoskeletal or mental disorders	3 months	Low	For participants with mental disorders and with musculoskeletal disorders there was no association between self-efficacy and RTW in multiple logistic regression analysis.
Waynor et al. 2016<br>United States<br>(North-America)	Prospective cohort study	N: 105<br>A: 44 (10.8)<br>G: 62 males, 43 females<br>O: Diverse<br>H: Serious mental illness	6 months	Moderate	Baseline self-efficacy was not a positive predictor of competitive employment at 6 months. Only the subscale work-related social skills self-efficacy (one subscale of self-efficacy) was negatively associated with current employment status (r = -.24, $p = .03$) in univariate analysis and was a significant predictor of obtaining employment in multivariate logistic regression analysis. Lower work-related social skills self-efficacy was a predictor of competitive employment. The subscales career planning self-efficacy, general work skills self-efficacy and job securing skills self-efficacy were not.

* RTW: Return to work

Table 4

Association between person-related factor 'motivation' and work participation

**First author, year, country (continent)**	**Study design**	**Population**<br>**N: Number of subjects**<br>**A: Age; mean age (SD), range**<br>**G: Gender**<br>**O: Occupation**<br>**H: Health status**	**Follow-up**	**Risk of bias**	**Results**
Åhrberg et al. 2010<br>Sweden<br>(Europe)	Qualitative study	N: 7<br>A: 30-57<br>G: 0 males, 7 females<br>O: -<br>H: Chronic pain problems	-	Moderate	Motivation was important for RTW according to women who were on long-term sick leave.
Awang et al. 2016<br>Malaysia<br>(Asia)	Cross-sectional study	N: 9850<br>A: <30: 3041, 30-39: 3081, >39: 3728<br>G: 7776 males, 2074 females<br>O: Diverse<br>H: Work-related injury	-	Moderate	Participants who successful returned to formal employment were more often motivated than participants with unsuccessful RTW ($p < .01$) in univariate Chi-square analysis. Motivated participants were 7 times more likely to return to employment compared to those who were not motivated (B: 2.04, OR: 7.67 (5.50-10.69), $p < .05$) in multivariate logistic regression analysis.
Boyle et al. 2014<br>Australia<br>(Oceania)	Cross-sectional study	Employed<br>N: 14<br>A: 36.5 (12.6), 18-75<br>G: 11 males, 3 females<br>O: Diverse<br>H: Spinal cord injury or traumatic brain injury<br>Unemployed<br>N: 16<br>A: 41.8 (13.7), 18-75<br>G: 15 males, 1 female<br>O: Diverse<br>H: Spinal cord injury or traumatic brain injury	-	Low	The most commonly cited facilitator for employment was motivation.
Braathen et al. 2007<br>Norway<br>(Europe)	Non-randomized experimental study	Intervention<br>N: 183<br>A: <40: 44, 40-49: 68, >49: 61<br>G: 48 males, 135 females<br>O: -<br>H: Long-term sick leave<br>Control<br>N: 96<br>A: <40: 25, 40-49: 31, >49: 36<br>G: 24 males, 72 females<br>O: -<br>H: Long-term sick leave	4 months	Moderate	RTW at 4 months was predicted by improved work motivation (B = .67, OR: 1.96 (1.04-3.69), $p = .04$) in multivariate logistic regression analysis. Improvement of one unit of a 5-point motivation scale increased the probability of RTW by 96% .

De Vries et al. 2011<br>The Netherlands<br>(Europe)	Qualitative study	N: 21<br>A: 49 (6.9), 10-60<br>G: 9 males, 12 females<br>O: Diverse<br>H: Chronic nonspecific musculoskeletal pain	-	Low	There were different important motivators for staying at work. Motivation to work may be considered to be an important prerequisite for staying at work.
Dekkers-Sánchez et al. 2010<br>The Netherlands<br>(Europe)	Qualitative study	N: 27<br>A: 49, 25-63<br>G: 14 males, 13 females<br>O: Blue collar, white collar<br>H: Chronic work disability	-	Moderate	Work motivation was a promoting factor for RTW proposed by work disabled participants.
Dunn et al. 2010<br>United States<br>(North-America)	Qualitative study	N: 23<br>A: 27-59<br>G: 11 males, 12 females<br>O: Diverse<br>H: Serious mental illness	-	Moderate	Having the motivation to work was important in helping participants with serious mental illness RTW and maintain their employment.
Elfving et al. 2009<br>Sweden<br>(Europe)	Prospective cohort study	N: 312<br>A: 43, 22-63<br>G: 144 males, 168 females<br>O: Diverse<br>H: Spinal pain	6 months	Low	Self-motivation was not a significant predictor of less sickness absence after 6 months in univariate analysis (logistic regression).
Hartke et al. 2011<br>United States<br>(North-America)	Qualitative study	N: 12<br>A: 51, 31-67<br>G: 8 males, 4 females<br>O: Diverse<br>H: Stroke	-	Moderate	Being motivated emerged to be an important facilitator that stroke survivors encounter in their efforts to RTW after stroke.
Lydell et al. 2011<br>Sweden<br>(Europe)	Prospective cohort study	N: 320<br>A: 42<br>G: 130 males, 190 females<br>O: Diverse<br>H: Musculoskeletal disorders	1, 5, 10 years	Moderate	There were more participants in the motivation and optimism group who had returned to work 1 year after baseline as compared to the hindrance and hesitation group. However, there were no significant differences between the groups after 5 and 10 years in univariate Chi-square analysis.
Puolakka et al. 2008<br>Finland<br>(Europe)	Prospective cohort study	N: 152<br>A: 39 (10)<br>G: 86 males, 66 females<br>O: -<br>H: Lumbar disc herniation	2 months, 5 years	Low	More motivation to work two months after surgery was associated with less work disability days after lumbar disc herniation surgery (B: -2.88 (-3.97- -1.79), $p < .001$). However, motivation for work was not associated with permanent disability pension after lumbar disc herniation in multivariate analysis.
Saperstein et al. 2011<br>United States<br>(North-America)	Cross-sectional study	N: 145<br>A: 42.76 (8.84)<br>G: 116 males, 29 females<br>O: -<br>H: Schizophrenia	-	Moderate	Intrinsic motivation was correlated with total hours worked after rehabilitation ($p = .01$) and mean work behavior ratings ($p < .001$) in univariate analysis. Intrinsic motivation was significantly correlated with total hours worked after rehabilitation ($\beta = 0.31$, $p = 0.002$) and mean work behavior ratings at the conclusion of rehabilitation ($\beta = 0.32$, $p = 0.001$) in hierarchical regression analysis. Intrinsic motivation was a significant mediator of the relationship between negative symptoms and work outcomes.
Van Velzen et al. 2011<br>The Netherlands<br>(Europe)	Qualitative study	N: 12<br>A: 52.1 (6.7), 18-60<br>G: 9 males, 3 females<br>O: Diverse<br>H: Brain injury	-	Moderate	Motivation was according to almost all participants a facilitator for RTW.
Wan Kasim et al.	Cross-	N: 126	-	Low	Being motivated to work was associated with successful employment ($p < .000$) in bivariate analysis. However, when this variable

2014<br>Malaysia<br>(Asia)	sectional study	A: 39.6 (9.2), 18-60<br>G: 113 males, 13 females<br>O: Diverse<br>H: Severe mental illness			was entered into a stepwise logistic regression model to predict successful employment status, motivation was not significant.
Wilbanks and Ivankova 2015<br>United States<br>(North-America)	Qualitative study	N: 4<br>A: 42-57<br>G: 3 males, 1 female<br>O: Diverse<br>H: Spinal cord injury	-	Low	Motivation was one of the four major themes that emerged as a facilitator for RTW from interviews with participants with spinal cord injury. Both extrinsic and intrinsic motivators seem to be important for rejoining the workforce.

* RTW: Return to work

Table 5

Association between person-related factor 'feelings of control' and work participation

**First author, year, country (continent)**	**Study design**	**Population**<br>**N: Number of subjects**<br>**A: Age; mean age (SD), range**<br>**G: Gender**<br>**O: Occupation**<br>**H: Health status**	**Follow-up**	**Risk of bias**	**Results**
Boot et al. 2008<br>The Netherlands<br>(Europe)	Cross-sectional study	Employed<br>N: 345<br>A: 44.2 (10.2)<br>G: 168 males, 177 females<br>O: -<br>H: Chronic physical disease<br>Fully work-disabled<br>N: 170<br>A: 52.4 (8.6)<br>G: 66 males, 104 females<br>O: -<br>H: Chronic physical disease	-	Low	The participants in the employed group had more positively oriented illness perceptions about the personal control over their illness ($p < .001$) compared to those who were fully work-disabled in univariate analysis, but personal control had no effect in multivariate analysis.
Busch et al. 2007<br>Sweden<br>(Europe)	Prospective cohort study	Sick Absent<br>N: 130<br>A: <45: 64, >45: 66<br>G: 45 males, 85 females<br>O: -<br>H: Chronic musculoskeletal pain<br>Work Capable<br>N: 103<br>A: <45: 58, >45: 45<br>G: 32 males, 71 females<br>O: -<br>H: Chronic musculoskeletal pain	3, 7, 11, 15 months	Low	Mastery (beliefs about control over current life and future) was a significant predictor of long-term sickness absence in univariate analysis ($p < .05$). Low sense of self mastery (B = .73, OR: 2.08 (1.27-3.40), $p = .004$) was a significant predictor of long-term sickness absence during follow-up in multivariate logistic regression analysis.
Dionne et al. 2013<br>Canada<br>(North-America)	Qualitative study	No RTW or recent RTW<br>N: 10<br>A: 30-39: 2, 40-49: 3, 50-59: 5, >59: 0<br>G: 7 males, 3 females<br>O: Manual work, service job<br>H: Back pain<br>RTW<br>N: 9<br>A: 30-39: 1, 40-49: 4, 50-59: 3, >59: 1	-	Moderate	The participants' perceptions of the control they had over their health problem played an important role in determining whether or not they returned to work. Participants in the no or recent return group considered that their return depended more on factors related to the work environment and to the healthcare system than on personal factors (extern instead of intern locus of control).

		G: 7 males, 2 females<br>O: Manual work, service job<br>H: Back pain			
Ekberg et al. 2015<br>Sweden<br>(Europe)	Prospective cohort study	RTW <3 months<br>N: 195<br>A: 44 (11), 18-65<br>G: 38 males, 157 females<br>O: 77 white collar, 89 pink collar, 29 blue collar<br>H: Common mental disorders<br>RTW 3-12 months<br>N: 98<br>A: 44 (10), 18-65<br>G: 14 males, 84 females<br>O: 47 white collar, 34 pink collar, 16 blue collar<br>H: Common mental disorders	1 year	Low	Mastery (the extent to which individuals perceive themselves in control of forces that affect their lives) was not different between the participants who returned within 3 months and the participants who returned in 3-12 months in univariate Chi-square analysis. Mastery was not related to RTW in the 3 month sub-cohort or in the 3-12 months sub-cohort in multivariate Cox regression analysis.
Karoly et al. 2013<br>United States<br>(North-America)	Cross-sectional study	On disability<br>N: 434<br>A: 25-44:122, 45-64: 260, 65-80: 52<br>G: 221 males, 213 females<br>O: -<br>H: Chronic pain<br>Working<br>N: 859<br>A: 25-44: 464, 45-64: 309, 65-80: 86<br>G: 481 males, 378 females<br>O: -<br>H: Chronic pain	-	Moderate	Participants who were working had higher scores on pain control than participants on disability ($p < .05$) in a t-test. Pain control was not a predictor of work status in multivariate logistic regression analysis.
Krause et al. 2013<br>Germany<br>(Europe)	Cross-sectional study	Early-retired<br>N: 39<br>A: 45.87 (9.57), 18-60<br>G: 15 males, 24 females<br>O: -<br>H: Multiple Sclerosis<br>Employed<br>N: 48<br>A: 34.96 (10.04), 18-60<br>G: 19 males, 29 females<br>O: -<br>H: Multiple Sclerosis	-	Moderate	Post-hoc analysis revealed that there are no significant differences between early retired participants with MS and employed participants with MS on self- reported health locus of control in univariate analysis.
Murphy et al. 2011<br>Australia<br>(Oceania)	Prospective cohort study	N: 72<br>A: 35.3 (14.4)<br>G: 58 males, 14 females<br>O: -	2 years	Moderate	Internal locus of control had a positive relationship with paid employment at 2 years ($p < .05$). However internal locus of control was not significant in predicting employment status in univariate analysis. Locus of control was not a significant predictor of employment in multivariate discriminant function analysis.

		H: Spinal cord injury			
Richard et al. 2011<br>Canada<br>(North America)	Prospective cohort study	N: 1007<br>A: 38.7 (10.6), 18-64<br>G: 589 males, 418 females<br>O: -<br>H: Back pain	2 years	Low	Participants who had a higher level of powerful others health locus of control were more likely to be found in the failure after attempt or in the failure group at 2 years in bivariate analyses ($p < .05$) in comparing to participants with a lower score. Participants with a higher level of chance health locus of control were more likely to be found in failure group at 2 years in bivariate analyses ($p < .05$) instead of participants with a lower score. There was no significant effect of internal locus of control on RTW in bivariate analysis. In multivariate analysis none of the forms of health locus of control was significant in predicting RTW.
Roesler et al. 2013<br>Australia<br>(Oceania)	Prospective cohort study	N: 192<br>A: 35.1, 18-63<br>G: 163 males, 29 females<br>O: Blue collar, white collar<br>H: Hand injury	4, 12 weeks	Low	Participants who attributed recovery to external factors at 28 days were more than five times more likely to have a delayed recovery (RTW later than 12 weeks) (B = 1.70, Exp (B) = 5.11, $p = .015$) in multivariate logistic regression analysis.
Selander et al. 2007<br>Sweden<br>(Europe)	Prospective cohort study	N: 347<br>A: 42<br>G: 187 males, 160 females<br>O: -<br>H: Back pain	6 months	Low	Participants with high internal locus of control had roughly 70% better chances of receiving a positive rehabilitation outcome (less sickness allowance than before) as compared to participants with low internal locus in bivariate ($p = .000$) and in multivariate analysis (OR: .73 (.59 - .89), $p = .003$).
Sluiter and Frings-Dresen 2008<br>The Netherlands<br>(Europe)	Cross-sectional study	Employed<br>N: 745<br>A: 40.0 (8.3)<br>G: 283 males,462 females<br>O: -<br>H: Repetitive strain injury<br>Sick-listed<br>N: 376<br>A: 42.3 (9.4)<br>G: 83 males, 293 females<br>O: -<br>H: Repetitive strain injury	-	Low	Participants with repetitive strain injury who were sick listed differed from the working group in in personal control ($p < .001$) in multivariate analysis of variance. Sick-listed participants scored lower on personal control than participants in the working group which indicated that they felt they had less control over their disease.
Torres et al. 2009<br>Spain<br>(Europe)	Prospective cohort study	N: 98<br>A: 43.6 (7.9), 21-59<br>G: 3 males, 95 females<br>O: Blue collar, white collar<br>H: Fibromyalgia	1, 3, 6, 12 months	Low	High scores on the fate scale (factor in the chance subscale) of the multidimensional health locus of control pain questionnaire predicted which participants with fibromyalgia would not be able to return after discharge (B = .102, $\chi^2 = 24.47$, Exp (B): 1.107 (1.06-1.15), $p < .001$) in the full model in multivariate logistic regression and which participants remained active during at least 6 months during follow up (B = .039, $\chi^2 = 15.37$, Exp (B): 1.039 (1.02-1.06), $p < .001$). However, for the last model was inadequate goodness-of-fit. Other scales of multidimensional health locus of control scale (internal, powerful professionals, luck subscales) did not have a significant effect.
Truchon et al. 2010<br>Canada<br>(North-America)	Prospective cohort study	N: 439<br>A: 38 (10), 18-60<br>G: 261 males, 178 females<br>O: -<br>H: Low back pain	6, 12 months	Moderate	At 12 months, cognitive appraisal of low back pain (control of pain and fear of physical activity) was predictive of absence from work (work status), when controlling for other variables (OR: 2.57, $p = .00$) in multivariate logistic regression analysis, but cognitive appraisal was not predictive for work absence at 6 months. Cognitive appraisal did also predict days of absence at 6 months ($\beta = 17.11$, $p < .05$) and at 12 months ($\beta = 38.72$, $p < .05$) in multivariate analysis.
Vlasveld et al. 2013<br>The Netherlands<br>(Europe)	Cross-sectional study	Current depressive or anxiety disorder<br>N: 1023<br>A: 40.6 (11.2), 18-65<br>G: 363 males, 660 females<br>O: -<br>H: Current depressive/anxiety disorder	-	Low	In participants with psychopathology external locus of control was associated with long-term absenteeism (longer than 2 weeks) (OR: .744 (.63-.89), $p = .001$), but not with short-term absenteeism (shorter than 2 weeks) in multinomial logistic regression analysis.

		Remitted depressive or anxiety disorder<br>N: 402<br>A: 43.3 (11.1), 18-65<br>G: 128 males, 274 females<br>O: -<br>H: Remitted depressive/anxiety disorder			
Volker et al. 2015<br>The Netherlands (Europe)	Prospective cohort study	N: 493<br>A: >44: 308 of 487<br>G: 220 males, 237 females<br>O: -<br>H: Long-term sick-listed	2 years	Low	Participants who scored high on sense of mastery (more control) had a shorter time to RTW compared to participants with low sense of mastery (HR: 1.668 (1.202-2.315), *p* = .002) in bivariate Cox regression analysis. Sense of mastery was not significantly associated with shorter duration until RTW in multivariate analysis.

* RTW: Return to work

Table 6

Association between person-related factor 'perceived health' and work participation

**First author, year, country (continent)**	**Study design**	**Population**<br>**N: Number of subjects**<br>**A: Age; mean age (SD), range**<br>**G: Gender**<br>**O: Occupation**<br>**H: Health status**	**Follow-up**	**Risk of bias**	**Results**
Audhoe et al. 2012<br>The Netherlands<br>(Europe)	Prospective cohort study	N: 932<br>A: 18-34: 232, 35-44: 260, 45-65: 418<br>G: 398 males, 514 females<br>O: Agency workers<br>H: Psychological complaints	10, 18, 27 months	Low	Moderate to good perceived health instead of poor health was associated with work participation (not being sick–listed and being able to or being back to work) at 18 months (*p* <. 01) and with work participation at 27 months (*p* < .01) in univariate analysis. Moderate to good perceived health (OR: 4.2 (2.43-7.20), *p* < .01) at 10 months was a prognostic factor for work participation at 18 months in multiple logistic analysis. Perceived health at 18 months was not a prognostic factor for work participation at 27 months in multiple logistic analysis.
Boot et al. 2014<br>Canada<br>(North-America)	Prospective cohort study	No comorbidity<br>N: 1382<br>A: 38.4 (10.9)<br>G: 700 males, 682 females<br>O: Diverse<br>H: Musculoskeletal injuries<br>Comorbidity<br>N: 183<br>A: 43.0 (11.7)<br>G: 94 males, 89 females<br>O: Diverse<br>H: Musculoskeletal injuries and somatic comorbidity	1 year	Low	A better general health was a significant predictor of RTW after 12 months for participants with and without comorbidity (OR: 1.77 (1.21-2.58)) in univariate logistic regression analysis.
Boot et al. 2011<br>The Netherlands<br>(Europe)	Cross-sectional study	N: 7748<br>A: 43 (12), 15-65<br>G: 3968 males, 3780 females<br>O: -<br>H: Chronic illness	-	Moderate	Lower perceived health status was associated with more sick leave in each chronic illness group (B > 0 ; *p* < .05) in multivariate analysis. Limitations at work, work characteristics and work adjustments partially explained the association between perceived health and sick leave.
Chen et al. 2012<br>Taiwan<br>(Asia)	Cross-sectional study	N: 120<br>A: 35.7, 16-23: 18, 25-45: 78, 46-60: 24<br>G: 92 males, 28 females<br>O: -<br>H: Work-related hand injury	-	Low	Self-perceived general health was not associated with time of work in multivariate stepwise regression analysis.
Dawson et al. 2011<br>Australia and New Zealand<br>(Oceania)	Cross-sectional study	No sick leave<br>N:1678<br>A: 45.2 (37.0-51.1), 18-65<br>G: 126 males, 1552 females<br>O: Nursing or midwives<br>H: Low back pain<br>Sick leave	-	Moderate	General physical health was better for participants who were not on sick leave than for participants who were on sick leave in bivariate analyses (OR: .94 (.93-.96), *p* < .001).

		N: 486<br>A: 43.9 (35.2-51.0), 18-65<br>G: 47 males, 439 females<br>O: Nursing or midwives<br>H: Low back pain			
De Vries et al. 2012b<br>The Netherlands<br>(Europe)	Cross-sectional study	Staying at work<br>N: 119<br>A: 51, 20-60<br>G: 48 males, 71 females<br>O: -<br>H: Chronic nonspecific musculoskeletal pain<br>Sick leave and referred for rehabilitation<br>N: 122<br>A: 39, 20-60<br>G: 56 males, 66 females<br>O: -<br>H: Chronic nonspecific musculoskeletal pain	-	Low	General health perception was not significantly different between participants who were staying at work and who were sick-listed in univariate analysis.
Dionne et al. 2007<br>Canada<br>(North-America)	Prospective cohort study	N: 1007<br>A: 38.7 (10.6), 18-64<br>G: 589 males, 418 females<br>O: -<br>H: Back pain	6 weeks, 12 weeks, 1 year, 2 years	Low	Fair or poor self-reported health status instead of excellent or very good health status was associated with failure in RTW in good health for men (OR: 8.20 (1.14–58.75), *p* < .05) and with partial success in RTW for men (OR: 3.08 (1.01–9.42), *p* < .05) but not with failure after attempts in multivariate analysis. The influence of general health on RTW for women was not studied.
Dyster-Aas et al. 2007<br>Sweden<br>(Europe)	Cross-sectional study	N: 48<br>A: 44.4 (10.2), 23-64<br>G: 37 males, 11 females<br>O: -<br>H: Burn injury	-	Low	Participants who returned to work scored higher on general health than participants who did not RTW (z = 2.9, *p* < .01) in a univariate Mann-Whitney U test.
Ekberg et al. 2015<br>Sweden<br>(Europe)	Prospective cohort study	RTW <3 months<br>N: 195<br>A: 44 (11), 18-65<br>G: 38 males, 157 females<br>O: 77 white collar, 89 pink collar, 29 blue collar<br>H: Common mental disorders<br>RTW 3-12 months<br>N: 98<br>A: 44 (10), 18-65<br>G: 14 males, 84 females<br>O: 47 white collar, 34 pink collar, 16 blue collar<br>H: Common mental disorders	1 year	Low	Participants who returned to work within 3 months had a higher self-rated health than participants who returned to work at 3-12 months (*p* = .005) in univariate Chi-square analysis. Self-rated health remained associated with RTW when looking at the health, function and work ability factors in the sub-cohort of participants returning within 3-12 months in multivariate Cox regression analysis, but not in the sub-cohort for participants who returned to work within 3 months. Significant variables from multiple Cox regression within each block of predictors (health, function and work ability, personal resources and work conditions) were included in the full model. Self-rated health was not associated with early RTW in the final model in multivariate Cox regression analysis.
Elfving et al. 2009<br>Sweden<br>(Europe)	Prospective cohort study	N: 312<br>A: 43, 22-63<br>G: 144 males, 168 females	6 months	Low	General health was not a significant predictor of less sickness absence after 6 months in univariate logistic regression analysis.

		O: Diverse<br>H: Spinal pain			
Grøvle et al. 2013<br>Norway<br>(Europe)	Prospective cohort study	Analysis RTW at 2 years<br>N: 237<br>A: 43.2 (9.2), >18<br>G: 136 males, 101 females<br>O: -<br>H: Sciatica<br>Analysis time to sustained RTW<br>N: 125<br>A: 42.1 (10.4), >18<br>G: 68 males, 57 females<br>O: -<br>H: Sciatica	2 years	Low	General health was associated with RTW at 2 years (*p* = .00) and time to sustained RTW (*p* = .01) in univariate analyses. General health was significantly associated with a higher chance of RTW at the 2-year follow-up in multivariate analysis (OR: 1.03 (1.01-1.05), *p* < .01), but not with time to sustained RTW.
Hansen et al. 2009<br>Denmark<br>(Europe)	Prospective cohort study	N: 75<br>A: 46 (10.1)<br>G: 22 males, 53 females<br>O: -<br>H: Endoscopic carpal tunnel release	3 months	Moderate	Self-reported health status was not a significant predictor of sick leave of more than 21 days in univariate logistic regression or multiple logistic regression.
He et al. 2010<br>China<br>(Asia)	Retrospective cohort study	N: 323<br>A: 37.8 (8.7), <60<br>G: 259 males, 64 females<br>O: Workers in a state-owned locomotive vehicles company<br>H: Occupational injury	7 months	Low	Participants with bad self-perceived health status instead of good perceived health status got less chance of successfully RTW in univariate (*p* < .01) and in multivariate Cox regression analysis (B: .32, HR: .35 (.18-.65), *p* < .001).<br>Participants with better self-report health status took shorter sick leave (*p* < .01) in univariate analysis.
Iakova et al. 2012<br>Switzerland<br>(Europe)	Prospective cohort study	N: 411<br>A: 43.3 (10.3), <60<br>G: 336 males,75 females<br>O: -<br>H: Orthopedic trauma	2 years	Moderate	General health at admission (*p* < .001), improvement in general health during stay (*p* = .02), and physical health (*p* = .002) were significant for predicting RTW at 2 years in univariate analysis, but not in the complete model in multivariate regression analysis. However, backward selection created a minimal model with a minimal amount of predictors to predict RTW and in this model improvement of general health (OR: 1.16 (1.04-1.28)) was a significant predictor of RTW.
Jensen et al. 2013<br>Denmark<br>(Europe)	Prospective cohort study	N: 325<br>A: 41.7 (10.4), 18-60<br>G: 159 males, 166 females<br>O: -<br>H: Low back pain	1 year	Low	Perceiving general health as bad (OR: 3.25 (1.53-6.89), *p* = .002) instead of very good, good or not so good, predicted unsuccessful RTW at after one year in univariate analysis, but did not contribute in the final model in multivariate analysis.
Lindell et al. 2010<br>Sweden<br>(Europe)	Prospective cohort study	Rehabilitation group<br>N: 63<br>A: 42.2 (2.4), <60<br>G: 30 males, 33 females<br>O: 87% blue collar<br>H: non-acute non-specific spinal pain<br>Primary-care group<br>N: 62<br>A: 43.0 (2.6), <60	6, 12, 18, 24 months	Low	State of health was positively associated with stable RTW at 6 (*p* = .02) and 18 months (*p* = .01) in univariate analysis, but was not a significant predictor in the multiple logistic regression.

		G: 27 males, 35 females<br>O: 87% blue collar<br>H: non-acute non-specific spinal pain			
Morrison et al. 2016<br>USA<br>(North America)	Prospective cohort study	Employed full-time<br>N: 219<br>A: 54.2 (11.6), 18-75<br>G: 132 males, 87 females<br>O: -<br>H: Cancer and hematopoietic stem cell transplantation<br>Underemployed because of health<br>N: 188<br>A: 53.3 (12.4), 18-75<br>G: 124 males, 64 females<br>O: -<br>H: Cancer and hematopoietic stem cell transplantation<br>Underemployed not because of health<br>N: 77<br>A: 56.5 (12.3), 19-74<br>G: 40 males, 37 females<br>O: -<br>H: Cancer and hematopoietic stem cell transplantation	1 year	Moderate	There was a significant association between 1 year employment and current perceived health ($\chi^2 = 91.24$, $p < .001$) in Chi-square analysis. Full-time employed participants reported their health as very good or good, those who were unemployed reported their health as good, fair or poor in univariate analysis.
Murgatroyd et al. 2016<br>Australia<br>(Oceania)	Prospective cohort study	N: 334<br>A: 36 (13.9), >18<br>G: 267 males, 67 females<br>O: Diverse<br>H: Upper and/or lower extremity fractures	6, 12, 24 months	Moderate	Self-assessed pre injury health status ($p = .03$) was associated with RTW in univariate analysis. Having fair to poor self-assessed pre-injury health status instead of having excellent health status was associated with longer time to RTW (HR: .36 (.14-.91), $p < .05$) in the Cox model in multivariate Cox regression analysis.
Nielsen et al. 2012<br>Denmark<br>(Europe)	Prospective cohort study	N: 205<br>A: 40.1 (10.1)<br>G: 42 males, 163 females<br>O: Private, governmental and municipal sector<br>H: Mental health problems	Max 52 weeks	Low	Self-rated health predicted a shorter time to RTW in univariate analysis ($p < .05$) and in multivariate analysis (HR: 1.18 (1.03-1.34), $p < .05$) with Cox regression modeling.
Ramel et al. 2013<br>Sweden<br>(Europe)	Prospective cohort study	RTW<br>N: 27<br>A: 38 (14.2), 19-64<br>G: 22 males, 5 females<br>O: -<br>H: Hand injury<br>No RTW	3, 12 months	Low	Participants who did not RTW after 3 months had a lower health status at 12 months than participants who did RTW ($p = .039$). Participants who did not RTW after 12 months had a lower self-reported health status at 12 months than participants who did RTW ($p = .023$) in univariate logistic regression analysis.

		N: 13<br>A: 42 (12.2), 19-64<br>G: 10 males, 3 females<br>O: -<br>H: Hand injury			
Richter et al. 2011<br>The Netherlands<br>(Europe)	Prospective cohort study	N: 276<br>A: 45 (7)<br>G: 256 males, 20 females<br>O: Self-employed<br>H: Musculoskeletal symptoms	1 year	Low	General health was not a prognostic factor for claim duration in univariate or multivariate analysis.
Sampere et al. 2012<br>Spain<br>(Europe)	Prospective cohort study	N: 663<br>A: 39.8 (11.4)<br>G: 364 males, 299 females<br>O: -<br>H: Musculoskeletal disorder, mental disorders or other physical conditions	2 years	Low	General health status was associated with time to RTW in bivariate analysis ($p < .05$). Poor perceived health status (HR: .71 (.59-.85)) instead of a good perceived health status was associated with a longer time to RTW in the full model among the total study population in multivariate analysis. Perceived poor health status was only significantly related to longer non-work related episodes in women (HR: .63 (.42-.95)) but not for men, when comparing men with women. Perceived health status was not related to longer time till RTW for workers on sick leave for mental disorders in multivariate analysis, but was related for participants with musculoskeletal disorders (HR: .52 (.39-.71)) and for participants with other physical conditions (HR: .72 (.52-.99)).
Sivertsen et al. 2013<br>Norway<br>(Europe)	Cross-sectional study	Work group<br>N: 2161<br>A: 51.29 (9.68), 20-69<br>G: 1067 males, 1094 females<br>O: -<br>H: Musculoskeletal pain<br>Sick leave group<br>N: 4511<br>A: 49.9 (9.95), 20-69<br>G: 1576 males, 2935 females<br>O: -<br>H: Musculoskeletal pain	-	Low	The work group and sick leave group differed in self-rated health status (higher for work group) ($p = .001$) in univariate analysis. Self-rated health was a significant predictor of work status (B = .51, Exp (B) = 1.66 (1.49-1.84), $p = .0001$) in multivariate logistic regression analysis. When self-rated health raised with one unit, the odds for being in the work group increased by 66%.
Vuistiner et al. 2015<br>Switzerland<br>(Europe)	Prospective cohort study	N: 1090<br>A: 42.9 (11.3), <62<br>G: 890 males, 200 females<br>O: -<br>H: Orthopaedic trauma	4 years	Low	The probability of being declared fit for work was higher in participants with better perceived general health at hospitalization in univariate and multivariate Cox regression analysis (HR: 1.16 (1.13-1.19)).

* RTW: Return to work

Table 7

Association between person-related factor 'coping strategies' and work participation

**First author, year, country (continent)**	**Study design**	**Population**<br>**N: Number of subjects**<br>**A: Age; mean age (SD), range**<br>**G: Gender**<br>**O: Occupation**<br>**H: Health status**	**Follow-up**	**Risk of bias**	**Results**
Arwert et al. 2017<br>The Netherlands<br>(Europe)	Cross-sectional study	RTW<br>N: 18<br>A: 48.5 (9.5), 20-90 (18-65 during hospitalization)<br>G: 10 males, 8 females<br>O: -<br>H: Stroke<br>Non-RTW<br>N: 28<br>A: 47.1 (9.9), 20-90 (18-65 during hospitalization)<br>G: 19 males, 9 females<br>O: -<br>H: Stroke	2-5 years	Low	Participants who returned to work scored lower on avoidance coping than participants who did not RTW in univariate ($p < .05$) and multivariate analysis (OR: .20 (.05–.78), $p < .05$). There were no significant differences in other types of coping (active coping and seeking support) in univariate and multivariate logistic regression analysis.
Becker et al. 2007<br>United States<br>(North-America)	Qualitative study	N: 38<br>A: 49.2 (7.0)<br>G: 22 males, 17 females<br>O: Diverse<br>H: Severe mental illness	-	Moderate	Appropriate skills for coping with psychiatric problems appeared to play an important role in finding and maintaining work for participants with severe mental illness.
Dawson et al. 2011<br>Australia and New Zealand<br>(Oceania)	Cross-sectional study	No sick leave<br>N:1678<br>A: 45.2 (37.0-51.1), 18-65<br>G: 126 males, 1552 females<br>O: Nursing or midwives<br>H: Low back pain<br>Sick leave<br>N: 486<br>A: 43.9 (35.2-51.0), 18-65<br>G: 47 males, 439 females<br>O: Nursing or midwives<br>H: Low back pain	-	Moderate	Participants on sick leave showed more passive coping ($p < .001$) than participants who were not on sick leave in univariate analysis. There was no difference in active coping in univariate analysis. Passive coping increased the likelihood of low back pain sick leave in the preceding 12 months (OR: 1.07 (1.04-1.12), $p < .001$) in multivariate analysis.
De Vries et al. 2011<br>The Netherlands<br>(Europe)	Qualitative study	N: 21<br>A: 49 (6.9), 10-60<br>G: 9 males, 12 females<br>O: Diverse<br>H: Chronic nonspecific musculoskeletal pain	-	Low	Coping with pain was according to participants an important factor for staying at work.

De Vries et al. 2012b<br>The Netherlands<br>(Europe)	Cross-sectional study	Staying at work<br>N: 119<br>A: 51, 20-60<br>G: 48 males, 71 females<br>O: -<br>H: Chronic nonspecific musculoskeletal pain<br>Sick leave and referred for rehabilitation<br>N: 122<br>A: 39, 20-60<br>G: 56 males, 66 females<br>O: -<br>H: Chronic nonspecific musculoskeletal pain	-	Low	Active coping (higher for stay at work group) an passive coping (higher in sick leave group) were associated with work status in univariate analysis (*p* = .001), but not in multivariate logistic regression analysis. The coping form expression of emotions was higher in the staying at work group (*p* = .049) in univariate analysis and coping self-statements was higher in the staying at work group (*p* = .042) in univariate analysis. However, these two forms of coping were not associated with work status in multivariate logistic regression analysis. Other forms of coping (palliative reaction, social support) were not associated in univariate and multivariate logistic regression analysis.
Dekkers-Sánchez et al. 2010<br>The Netherlands<br>(Europe)	Qualitative study	N: 27<br>A: 49, 25-63<br>G: 14 males, 13 females<br>O: Blue collar, white collar<br>H: Chronic work disability	-	Moderate	Poor coping style was according to disabled participants a perpetuating factor for long-term sick leave. Coping was also a promoting factor for RTW proposed by work disabled participants.
Grytten et al. 2017<br>Norway<br>(Europe)	Prospective cohort study	Employed<br>N: 41<br>A: 32.29 (7.90)<br>G: 19 males, 22 females<br>O: Diverse<br>H: Multiple sclerosis<br>Unemployed<br>N: 52<br>A: 34.59 (10.14)<br>G: 10 males, 42 females<br>O: Diverse<br>H: Multiple sclerosis	13 years	Low	Unemployed and employed participants did not differ in coping style at baseline (planning, restraint coping, seeking social support for instrumental reasons, seeking social support for emotional reasons, positive reinterpretation and growth, focus on and venting of emotions, denial, behavioral disengagement, mental disengagement). Only the coping styles focus on and venting of emotions and denial were associated with time to awarding disability in univariate analysis (*p* = .05). For participants who were employed at baseline the coping strategy of denial was associated with shorter time to awarding disability pension (or a shorter time to unemployment) in multivariate analysis (HR: 1.59, 1.08-2.32, *p* = .02). Other coping styles were not significantly associated with time to awarding disability in multivariate analysis.
Hartke et al. 2011<br>United States<br>(North-America)	Qualitative study	N: 12<br>A: 51, 31-67<br>G: 8 males, 4 females<br>O: Diverse<br>H: Stroke	-	Moderate	Positive coping strategies was a category that emerged to be an important facilitator that stroke survivors encounter in their efforts to RTW after stroke.
Heymans et al. 2009<br>The Netherlands<br>(Europe)	Prospective cohort study	N: 628<br>A: 40.6 (9.5), 18-65<br>G: 446 males, 182 females<br>O: Blue collar, white collar workers<br>H: Low back pain	1 year	Low	Pain coping was not associated with work absence in multivariate Cox regression analysis.
Huijs et al. 2012<br>The Netherlands<br>(Europe)	Prospective cohort study	N: 682<br>A: 46.6 (9.25)<br>G: 284 males, 389 females<br>O: -	2 years	Low	A lower level of active problem solving coping was associated with a longer duration until full RTW for the group with physical problems and the group with mental problems (*p* < .05), but not for the group with both physical and mental problems in univariate analysis. However, multivariate analysis showed that the lower level of active problem solving coping was only a predictor of a longer duration until RTW in participants with both physical and mental problems (HR: 1.58 (1.04-2.41), *p* = .03). More avoidance

		H: Physical problems, mental problems or a combination of physical and mental problems			coping was a significant predictor of longer duration until full RTW in univariate analysis for participants with physical problems, but not for participants with mental problems or with both physical and mental problems. Avoidance coping was not a significant predictor in multivariate analysis in all three groups.
Iakova et al. 2012<br>Switzerland<br>(Europe)	Prospective cohort study	N: 411<br>A: 43.3 (10.3), <60<br>G: 336 males,75 females<br>O: -<br>H: Orthopedic trauma	2 years	Moderate	Participants who used more avoidance of situations that are reminders of the accident had lower chances of RTW in univariate analysis (*p* = .01) and in the complete model (OR: .69 (.61-.79), *p* < .001) in multivariate analysis.
Karoly et al. 2013<br>United States<br>(North-America)	Cross-sectional study	On disability<br>N: 434<br>A: 25-44:122, 45-64: 260, 65-80: 52<br>G: 221 males, 213 females<br>O: -<br>H: Chronic pain<br>Working<br>N: 859<br>A:25-44: 464, 45-64: 309, 65-80: 86<br>G: 481 males, 378 females<br>O: -<br>H: Chronic pain	-	Moderate	Participants who were working had higher scores on ignoring, task persistence and positive self-talk than participants who were not working in univariate analysis. Participants on disability had higher scores on guarding in a t-test. Task persistence was also associated with work status in multivariate analysis (b = .014, e^b: 1.15 (1.11-1.19), *p* < .001).
Luk et al. 2010<br>Hong Kong<br>(Asia)	Prospective cohort study	Returned to work<br>N: 28<br>A: 37 (9), 20-56<br>G: 57 males, 8 females<br>O: -<br>H: Chronic low back pain<br>Not returned to work<br>N: 26<br>A: 42 (8), 20-56<br>G: 22 males, 4 females<br>O:-<br>H: Chronic low back pain	7, 14 weeks, 6 months	Low	The pain control (ability to cope with pain) ratings did not significantly differ between participants who returned to work and participants who did not RTW in a t-test.
Lundqvist and Samuelsson 2012<br>Sweden<br>(Europe)	Qualitative study	N: 14<br>A: 51 (8.1), 37-63<br>G: 8 males, 6 females<br>O: -<br>H: Brain injury	-	Moderate	Coping (gaining awareness and develop coping strategies) was important for progressing in rehabilitation and RTW according to participants after acquiring brain injury.
Norlund et al. 2011<br>Sweden<br>(Europe)	Prospective cohort study	N: 117<br>A: 24-55<br>G: 35 males, 82 females<br>O: -<br>H: Burnout	1 year, 2 years	Low	Using covert coping towards supervisors or workmates was not associated with changes in sick leave in univariate analysis. However, using covert coping (choosing avoidance behavior when experiencing a conflict) towards supervisors (OR: 2.78 (1.17-6.62), *p* < .05) and workmates (OR: 2.58 (1.05-6.34), *p* < .05) was associated with unchanged sick leave, when adjusting for background characteristics in multivariate analysis. Participants who showed less covert coping, had less sick leave at follow-up in comparing to the days of sick leave at baseline in logistic regression analyses.
Øyeflaten et al. 2008<br>Norway	Prospective cohort study	N: 135<br>A: 45 (8.4), 24-61	3, 12 months	Low	The chances of RTW were higher with high scores on instrumental mastery-oriented coping than with low scores of coping at 3 months (OR: .3 (.10-.74), *p* = .01) and 12 months (OR: 5.9 (1.63-21.41), *p* = .007) in univariate and multivariate analysis. However

(Europe)		G: 48 males, 87 females<br>O: Diverse<br>H: Musculoskeletal complaints			other forms of coping (emotion-focused coping, hopelessness and coping expectancy) did not predict RTW in univariate or multivariate logistic regression analysis.
Øyeflaten et al. 2016<br>Norway<br>(Europe)	Prospective cohort study	N: 1155<br>A: 46 (9.1)<br>G: 349 males, 806 females<br>O: -<br>H: Long-term sick leave	3 years and 4 months	Moderate	Poor coping ability (r = .13, *p* <.001) was positively associated with days on sickness benefits after work rehabilitation in univariate analysis. Poor coping ability did not have a direct or an indirect effect on days on sickness benefits after work rehabilitation in multivariate analysis with structural equation modeling.
Strober and Arnett 2016<br>United States<br>(North-America)	Cross-sectional study	Unemployed<br>N: 27<br>A: 51.74 (8.31)<br>G: 0 males, 27 females<br>O: -<br>H: Multiple sclerosis<br>Employed<br>N: 41<br>A: 46.07 (8.93)<br>G: 0 males, 41 females<br>O: -<br>H: Multiple sclerosis	-	Moderate	Participants who left the workforce were more likely to endorse maladaptive coping behaviors like behavioral disengagement (F = 4.30, *p* = .042) and substance use (F = 6.04, *p* = .017) than participants who continued to work in multivariate analysis of covariance. There were no differences in the other 13 coping subscales.
Tamminga et al. 2012<br>The Netherlands<br>(Europe)	Qualitative study	N: 12<br>A: 42 (7), 18-65<br>G: 0 males, 12 females<br>O: -<br>H: Breast cancer	-	Low	The implementation of copings strategies to deal with cancer and work was a facilitator for RTW according to participants with breast cancer.
Truchon et al. 2010<br>Canada<br>(North-America)	Prospective cohort study	N: 439<br>A: 38 (10), 18-60<br>G: 261 males, 178 females<br>O: -<br>H: Low back pain	6, 12 months	Moderate	Avoidance coping did not predict employment status or days of absence in multivariate linear regression analysis.

* RTW: Return to work

Table 8

Association between person-related factor 'fear-avoidance beliefs' and work participation

**First author, year, country (continent)**	**Study design**	**Population**<br>**N: Number of subjects**<br>**A: Age; mean age (SD), range**<br>**G: Gender**<br>**O: Occupation**<br>**H: Health status**	**Follow-up**	**Risk of bias**	**Results**
Besen et al. 2015<br>United States<br>(North-America)	Prospective cohort study	N: 241 participants<br>A: 38 (11.4), 18-63<br>G: 130 males, 111 females<br>O: 76% blue collar<br>H: Low back pain	1 week, 3 months	Moderate	Fear-avoidance beliefs were positively related to days of absence (r = .21, $p < .001$) and negatively with work status (r = -.27, $p < .001$) in univariate analysis (correlations). In multivariate analysis fear-avoidance beliefs were only indirectly associated to days of absence and work status.
Carriere et al. 2015a<br>Canada<br>(North-America)	Prospective cohort study	N: 154<br>A: 36.4 (9.2), 20-60<br>G: 81 males, 73 females<br>O: Diverse<br>H: Whiplash associated disorders	1 year	Low	Participants who returned to work scored lower on fear-avoidance beliefs for movement than participants who did not RTW (t = 3.38, $p < .001$) in univariate analysis. Lower scores on fear-avoidance beliefs for movement (β = –.30, t(254) = –1.26, $p = .21$) were not associated with successful RTW when controlling for RTW expectations. Expectations mediated the relation between fear-avoidance beliefs for movement and RTW status in multivariate regression analysis.
Coggon et al. 2013<br>Diverse<br>(Europe, South-North-America, South-America, Africa, Asia, Oceania)	Cross-sectional study	N: 12416<br>A: 20-29: 3058, 30-39: 3971, 40-49: 3451, 50-59: 1936<br>G: 4348 males, 8068 females<br>O: Diverse<br>H: Musculoskeletal illness	-	Moderate	Fear-avoidance for physical activity was a significant predictor of prolonged sickness in the full model (PRR: .68 (.53-.85)) in multivariate Poisson regression analysis.
Dawson et al. 2011<br>Australia and New Zealand<br>(Oceania)	Cross-sectional study	No sick leave<br>N:1678<br>A: 45.2 (37.0-51.1), 18-65<br>G: 126 males, 1552 females<br>O: Nursing or midwives<br>H: Low back pain<br>Sick leave<br>N: 486<br>A: 43.9 (35.2-51.0), 18-65<br>G: 47 males, 439 females<br>O: Nursing or midwives<br>H: Low back pain	-	Moderate	Participants on sick leave had more fear-avoidance beliefs for movement ($p < .001$) than participants who were not on sick leave in bivariate analysis. Fear of movement was associated with sick leave in multivariate analyses for women (OR: 1.05 (1.02-1.08), $p = .001$) and for men (OR: 1.17 (1.05-1.29), $p = .004$).
De Vries et al. 2012b<br>The Netherlands<br>(Europe)	Cross-sectional study	Staying at work<br>N: 119<br>A: 51, 20-60<br>G: 48 males, 71 females	-	Low	Fear-avoidance for movement (higher in sick leave group) was negatively associated with work status in univariate analysis ($p = .001$).<br>Participants who were on sick leave scored higher on fear-avoidance beliefs for movement than participants who stayed at work (B = -.06, OR: .94 (.90-.99), $p = .028$) in multivariate logistic regression analysis. However, fear-avoidance beliefs for movement was

		O: -<br>H: Chronic nonspecific musculoskeletal pain<br>Sick leave and referred for rehabilitation<br>N: 122<br>A: 39, 20-60<br>G: 56 males, 66 females<br>O: -<br>H: Chronic nonspecific musculoskeletal pain			not one of the factors which best discriminated participants within the sick leave group and the staying at work group in backwards stepwise logistic regression analysis.
Dionne et al. 2007<br>Canada<br>(North-America)	Prospective cohort study	N: 1007<br>A: 38.7 (10.6), 18-64<br>G: 589 males, 418 females<br>O: -<br>H: Back pain	6 weeks, 12 weeks, 1 year, 2 years	Low	Fear-avoidance beliefs towards work were associated with failure in RTW in good health for women (OR: 3.01 (1.14–7.91), *p* <. 05) and for men (OR: 4.08 (1.76–9.44), *p* < .05) in multivariate analysis. Fear-avoidance beliefs for work were not associated with partial success of RTW or failure after attempt to RTW.<br>Fear-avoidance beliefs for activity were associated with partial success of RTW (OR: 1.36 (1.07-1.75), with failure after attempt (OR: 1.94 (1.27-2.95)) and with failure to RTW (OR: 1.98 (1.01-3.89)) for women.
Du Bois et al. 2009<br>Belgium<br>(Europe)	Prospective cohort study	N: 346<br>A: 41<br>G: 183 males, 163 females<br>O: 74% blue collar<br>H: Low back pain	6 months	Low	Fear-avoidance beliefs for work, fear-avoidance beliefs for activity and fear-avoidance beliefs for movement were associated with sick leave for longer than 3 months (*p* < .01) in univariate analysis. Only the item “It is not advisable to be physically active” for measuring fear-avoidance beliefs for movement was significantly associated with no RTW in multivariate analysis (OR: 1.39 (1.10-1.76)).
Dyster-Aas et al. 2007<br>Sweden<br>(Europe)	Cross-sectional study	N: 48<br>A: 44.4 (10.2), 23-64<br>G: 37 males, 11 females<br>O: -<br>H: Burn injury	-	Low	The participants who returned to work expressed less fear-avoidance beliefs (z = 3.2, *p* < .01) than those who were not working in a univariate Mann-Whitney U test.
Elfving et al. 2009<br>Sweden<br>(Europe)	Prospective cohort study	N: 312<br>A: 43, 22-63<br>G: 144 males, 168 females<br>O: Diverse<br>H: Spinal pain	6 months	Low	Fear-avoidance beliefs for movement were not a significant predictor of less sickness absence after 6 months in univariate logistic regression analysis.
Grøvle et al. 2013<br>Norway<br>(Europe)	Prospective cohort study	Analysis RTW at 2 years<br>N: 237<br>A: 43.2 (9.2), >18<br>G: 136 males, 101 females<br>O: -<br>H: Sciatica<br>Analysis time to sustained RTW<br>N: 125<br>A: 42.1 (10.4), >18<br>G: 68 males, 57 females<br>O: -<br>H: Sciatica	2 years	Low	Fear-avoidance beliefs for work were associated with RTW at 2 years (*p* = .00) and time to sustained RTW (*p* = .01) in univariate analyses. Less fear-avoidance for work was significantly associated with a higher probability of RTW at the 2-year follow-up (OR: .93, (.90-.97), *p* = .00) and more fear-avoidance for work was associated with a longer time to sustained RTW (OR: .97 (.95-.99), *p* = .00) in multivariate analysis.<br>Fear-avoidance beliefs for movement were associated with RTW at 2 years (*p* = .00), but not with time to sustained RTW in univariate analyses.
Heymans et al. 2009<br>The Netherlands<br>(Europe)	Prospective cohort study	N: 628<br>A: 40.6 (9.5), 18-65<br>G: 446 males, 182 females	1 year	Low	A higher score for fear-avoidance beliefs was associated with a higher risk for not returning to work at 6 months in multivariate Cox regression analysis (B: -.02, HR: .98 (.97-1.00)). However, the effect was small.<br>Fear-avoidance beliefs for movement were not associated with work absence in multivariate analysis (Cox regression).

		O: Blue collar, white collar<br>H: Low back pain			
Heymans et al. 2007<br>Australia<br>(Oceania)	Retrospective cohort study	N: 194<br>A: 41.8 (9.9), 18-65<br>G: 65 males, 129 females<br>O: -<br>H: Low back pain	6 months	Low	Less fear-avoidance beliefs were predictive for being returned to work at 6 months (OR: .95 (.91-.99)) in multivariate logistic regression analysis.
Jensen et al. 2013<br>Denmark<br>(Europe)	Prospective cohort study	N: 325<br>A: 41.7 (10.4), 18-60<br>G: 159 males, 166 females<br>O: -<br>H: Low back pain	1 year	Low	Fear-avoidance was associated with unsuccessful RTW (OR: 1.62 (1.27-2.06), *p* < .001) in univariate analysis, but did not contribute in the final model in multivariate analysis.
Karels et al. 2010<br>The Netherlands<br>(Europe)	Prospective cohort study	N: 483<br>A: 41.5 (10.4)<br>G: 161 males, 322 females<br>O: Diverse<br>H: Arm, neck and shoulder complaints	3, 6 months	Moderate	High fear-avoidance beliefs for movement were associated with the occurrence of sickness absence in univariate analysis (OR: 2.1 (1.3-3.4), *p* < .05), but not in multivariate analysis because it was correlated with other factors as somatization and catastrophizing.
Kovacs et al. 2007<br>Spain<br>(Europe)	Prospective cohort study	No sick leave<br>N: 77<br>A: 47<br>G: 31 males, 46 females<br>O: -<br>H: Low back pain<br>Short/medium sick leave<br>N: 46<br>A: 46<br>G: 21 males, 25 females<br>O: -<br>H: Low back pain<br>Long sick leave<br>N: 42<br>A: 45<br>G: 24 males, 18 females<br>O: -<br>H: Low back pain	1 year	Low	Scores on fear-avoidance for work, fear-avoidance for physical activity and total fear-avoidance were significantly lower for participants with no sick leave in comparing to participants with sick leave (*p* = .000) in univariate analysis. More total fear-avoidance beliefs increased the odds of being on sick leave for up to 60 days (OR: 1.02 (1.01-1.04), *p* = .011) and the odds of being sick listed for 61 to 365 days (OR: 1.08 (1.05-1.11), *p* = .000). When the subscales were used instead of the total fear-avoidance beliefs scale, only fear-avoidance for work was significant for predicting sick leave for up to 60 days (OR: 1.04 (1.01-1.08), *p* = .022) and sick leave for up to 61-365 days (OR: 1.11 (1.06-1.16), *p* = .000), but not fear-avoidance beliefs for physical activity. Each additional point in the FAB-Work score increased the odds of being on sick leave for up to 60 days by 4.2% and the odds of being sick listed for 61 to 365 days by 11.0% in a multivariate ordinal logistic regression model.
Magnussen et al. 2007b<br>Norway<br>(Europe)	Randomized controlled trial	Intervention<br>N: 45<br>A: 49.1 (6.4), 36-56<br>G: 19 males, 26 females<br>O: -<br>H: Back pain<br>Control<br>N: 44<br>A: 49 (4.5), 36-56<br>G: 14 males, 30 females<br>O: -	1 year	Moderate	There was no significant difference in fear-avoidance beliefs for work and fear-avoidance beliefs for activity between participants who had entered a RTW process (being on educational course or being in work training) and who had not entered a RTW process in univariate analysis. However, when adjusting for age and gender, the odds ratio for fear-avoidance for work was significant (OR = 10.6 (1.5-78.1), *p* < .05) in multivariate analysis, in the way that disability pensioners with a lower score on fear-avoidance were more likely to have entered a RTW process at 1-year follow-up.

		H: Back pain			
Mannion et al. 2009<br>Switzerland<br>(Europe)	Cross-sectional study	N: 670<br>A: 44.5 (10.2)<br>G: 362 males, 308 females<br>O: -<br>H: Low back pain	-	Moderate	Fear-avoidance beliefs for work and activity were higher for participants with work absence, than for participants without work absence (*p* = .0001) in univariate analysis with Spearman rank correlation coefficients. Fear-avoidance for work was a significant predictor of days of work absence (β = .15, *p* = .001) in hierarchical multiple regression analysis, but fear-avoidance for activity was not. Fear-avoidance beliefs for work and activity were also significantly different between participants with reductions in productivity and participants without reductions in productivity (*p* = .0001) in univariate analysis. Fear-avoidance for work was a significant predictor of reduced productivity at work due to low back pain (β = .26, *p* = .0001) in multivariate analysis, but fear-avoidance for activity was not.
Morris and Watson 2011<br>Jersey<br>(Europe)	Cross sectional study	Sick-listed<br>N: 62<br>A: 45.19 (10.41), >18<br>G: 40 males, 22 females<br>O: Private sector: 45, public: sector: 17<br>H: Low back pain<br>Non-sick-listed<br>N: 57<br>A: 46.04 (11.98), >18<br>G: 29 males, 28 females<br>O: Private sector: 45, public sector: 11<br>H: Low back pain	-	Low	Sick-listed participants recorded higher scores on the fear-avoidance for work measure (*p* < .000) than non- sick-listed participants in univariate analysis. There was no difference for fear-avoidance beliefs for activity. Logistic regression analysis revealed that fear-avoidance for work was a significant predictor of being sick-listed (B = .07, Exp (B) = 1.08 (1.02-1.13), *p* = .004) in multivariate analysis.
Opsahl et al. 2016<br>Norway<br>(Europe)	Prospective cohort study	N: 574<br>A: 44.3 (9.7), 20-60<br>G: 285 males, 289 females<br>O: -<br>H: Low back pain	1 year	Low	Low fear-avoidance beliefs for work were associated with more chance to RTW at 12 months for men and women in bivariate analysis in comparing to high scores of fear-avoidance beliefs (*p* < .05). Having moderate fear-avoidance beliefs for work instead of low fear-avoidance beliefs was associated with more chance to RTW at 12 months for women (*p* < .05), but not for men in bivariate analysis.
Øyeflaten et al. 2008<br>Norway<br>(Europe)	Prospective cohort study	N: 135<br>A: 45 (8.4), 24-61<br>G: 48 males, 87 females<br>O: Diverse<br>H: Musculoskeletal complaints	3, 12 months	Low	Fear-avoidance for work was the most important risk factor for not returning to work at 3 months (OR: 3.8 (1.30-11.32), *p* = .02) and 12 months (OR: 9.5 (2.40-37.53), *p* = .001) in univariate and multivariate analysis, but fear-avoidance for activity was not a risk factor.
Øyeflaten et al. 2016<br>Norway<br>(Europe)	Prospective cohort study	N: 1155<br>A: 46 (9.1)<br>G: 349 males, 806 females<br>O: -<br>H: Long-term sick leave	3 years and 4 months	Moderate	More fear-avoidance beliefs (r = .38, *p* <.001) were positively associated with days on sickness benefits after work rehabilitation (*p* <.001) in univariate analysis. Fear-avoidance beliefs for work were directly associated with days on sickness benefits after work rehabilitation (β = .27, *p* < .001) in the full structural model in multivariate analysis with structural equation modeling. Fear-avoidance beliefs for activity were not associated with RTW in univariate analysis.
Poulain et al. 2010<br>France<br>(Europe)	Prospective cohort study	N: 105<br>A: 32.9 (9.8)<br>G: 45 males, 60 females<br>O: Diverse<br>H: Chronic low-back pain	1, 6, 12 months, 3.5 years	Low	Participants who returned to work had less fear-avoidance beliefs for work (*p* = .01) and less global fear-avoidance beliefs (*p* = .05) than participants who had not returned to work in univariate analysis. Fear-avoidance beliefs for activity did not differ between the two groups. However, no form of fear-avoidance beliefs was an independent factor associated with RTW in a multivariate analysis.
Richter et al. 2011<br>The Netherlands<br>(Europe)	Prospective cohort study	N: 276<br>A: 45 (7)<br>G: 256 males, 20 females<br>O: Self-employed<br>H: Musculoskeletal symptoms	1 year	Low	Fear-avoidance beliefs for movement were associated with a longer period of claim duration in univariate analysis (*p* = .03), but not in multivariate Cox regression analysis.

Spector et al. 2012<br>United States<br>(North-America)	Prospective cohort study	N: 670<br>A: 44.9 (9.6), >18<br>G: 255 males, 415 females<br>O: Diverse<br>H: Carpal tunnel syndrome	2 years	Low	High or very high fear-avoidance for work was associated with higher odds of long-term disability (missing work days and retrieving compensation) in univariate analysis ($p < .05$) in comparing to having very low or moderate fear-avoidance. However, fear-avoidance was not a significant predictor of long-term disability in the final multi-domain model in multivariate analysis.
Steenstra et al. 2010<br>Canada<br>(North America)	Cross-sectional study	N: 442<br>A: 42.1 (10.3), >15<br>G: 254 males, 188 females<br>O: -<br>H: Low back pain	-	Low	Fear-avoidance beliefs for work and activity as measured 4 week after injury were not significantly associated with employment status at 4 weeks after injury in univariate Cox regression analysis.
Truchon et al. 2012<br>Canada<br>(North-America)	Prospective cohort study	N: 535<br>A: 42 (10), 18-60<br>G: 317 males, 218 females<br>O: Diverse<br>H: Low back pain	6, 12 months	High	Fear-avoidance beliefs for work ($\beta = -.35$, $p < .00$) were predictive of long-term absence from work in multivariate Cox regression analysis.
Turner et al. 2008<br>United States<br>(North-America)	Prospective cohort study	N: 1885<br>A: 39.4 (11.2)<br>G: 1282 males, 603 females<br>O: Diverse<br>H: Back injury	1 year	Low	Work fear-avoidance was associated with work disability ($p < .001$) in univariate analysis. In a multi-domain model none of the psychological factors contributed independently to the prediction of 1-year work disability (wage replacement compensation for total disability 12 months after claim submission). This was because the score on the Roland-Morris disability questionnaire (RDQ) was substantially correlated with all the psychological factors, including work fear-avoidance.

* RTW: Return to work

Table 9

Association between person-related factor ‘perceived work-relatedness’ and work participation

First author, year, country (continent)	Study design	Population<br>N: Number of subjects<br>A: Age; mean age (SD), range<br>G: Gender<br>O: Occupation<br>H: Health status	Follow-up	Risk of bias	Results
Coggon et al. 2013<br>Diverse<br>(Europe, South-North-America, South-America, Africa, Asia, Oceania)	Cross-sectional study	N: 12416<br>A: 20-29: 3058, 30-39: 3971, 40-49: 3451, 50-59: 1936<br>G: 4348 males, 8068 females<br>O: Diverse<br>H: Musculoskeletal illness	-	Moderate	The risk of prolonged sickness was higher for participants with adverse beliefs about work-relatedness of musculoskeletal pain (PRR 1.22 (1.01-1.47)) in Poisson regression models. However, when number of painful anatomical sites was included in the model, the effect of beliefs about work-relatedness of musculoskeletal pain was not significant anymore.
Dawson et al. 2011<br>Australia and New Zealand<br>(Oceania)	Cross-sectional study	No sick leave<br>N:1678<br>A: 45.2 (37.0-51.1), 18-65<br>G: 126 males, 1552 females<br>O: Nursing or midwives<br>H: Low back pain<br>Sick leave<br>N: 486<br>A: 43.9 (35.2-51.0), 18-65<br>G: 47 males, 439 females<br>O: Nursing or midwives<br>H: Low back pain	-	Moderate	People on sick leave perceived work more often as a cause for their pain ($p < .001$) in bivariate analysis, but perceiving work as a cause was not related to sick leave in multivariate analysis.
Jensen et al. 2013<br>Denmark<br>(Europe)	Prospective cohort study	N: 325<br>A: 41.7 (10.4), 18-60<br>G: 159 males, 166 females<br>O: -<br>H: Low back pain	1 year	Low	Blaming the work for pain (OR: 2.40 (1.40-4.12), $p = .002$) predicted unsuccessful RTW after one year in univariate analysis. Blaming the work for pain was in combination with other variables also negatively associated with RTW in multivariate logistic regression analyses ($p < .001$).
Karels et al. 2010<br>The Netherlands<br>(Europe)	Prospective cohort study	N: 483<br>A: 41.5 (10.4)<br>G: 161 males, 322 females<br>O: Diverse<br>H: Arm, neck and shoulder complaints	3, 6 months	Moderate	Self-reported work relatedness of complaints was significantly related to sickness absence during follow-up in the univariate model ($p < .05$), but also in the multivariate model (OR: 3.2 (1.6-6.4), $p < .05$).
Kuijer et al. 2016<br>The Netherlands<br>(Europe)	Retrospective cohort study	Returned to work<br>N: 121<br>A: 58.8 (8.3), <60: 69, >59: 52<br>G: 63 males, 58 females	At least 2 years	Low	Participant-reported work-relatedness of the knee symptoms was associated with no RTW after total knee arthroplasty surgery (OR: 5.3 (2.0-14.1)) in multivariate backward stepwise logistic regression.

		O: -<br>H: Knee arthroplasty<br>No RTW<br>N: 46<br>A: 62.1 (8.3), <60: 16, >59: 30<br>G: 19 males, 27 females<br>O: -<br>H: Knee arthroplasty			
Sampere et al. 2012<br>Spain<br>(Europe)	Prospective cohort study	N: 663<br>A: 39.8 (11.4)<br>G: 364 males, 299 females<br>O: -<br>H: Musculoskeletal disorder, mental disorders or other physical conditions	2 years	Low	Thinking that there was a partial or complete relation between health and job instead of making no connection was associated with a longer time to RTW in bivariate analysis ($p < .05$). However, thinking that there was a partial or complete connection between the current health problem and job was not significantly associated with time to RTW in the total study population in multivariate analysis. When looking in women and men apart, thinking that there was a complete relation between health an job was related to longer time to RTW for women (HR:.63 (.42-.95)). Making a partial connection (HR: .43 (.23-.81)) or making a complete connection (HR: .41 (.20-.82)) between health and job was related to longer time till RTW for participants on sick leave for mental disorders, but not for participants with musculoskeletal disorders or other physical conditions.
Turner et al. 2008<br>United States<br>(North-America)	Prospective cohort study	N: 1885<br>A: 39.4 (11.2)<br>G: 1282 males, 603 females<br>O: Diverse<br>H: Back injury	1 year	Low	Blame for injury (work, self, someone else or nothing) was not associated with work disability (wage replacement compensation for total disability 12 months after claim submission) in univariate analysis.

* RTW: Return to work

Table 10

Association between person-related factor 'catastrophizing' and work participation

**First author, year, country (continent)**	**Study design**	**Population**<br>**N: Number of subjects**<br>**A: Age; mean age (SD), range**<br>**G: Gender**<br>**O: Occupation**<br>**H: Health status**	**Follow-up**	**Risk of bias**	**Results**
Adams et al. 2017<br>Canada<br>(North-America)	Prospective cohort study	N: 80<br>A: males 46.7 (9.5), females 45.7 (8.3)<br>G: 26 males, 54 females<br>O: -<br>H: Major depressive disorder	1 month	Low	Reductions in catastrophizing were associated with a higher probability of occupational re-engagement (β = .36, OR: 1.4 (1.09-1.91), *p* = .01) in multivariate logistic regression analysis.
Besen et al. 2015<br>United States<br>(North-America)	Prospective cohort study	N: 241 participants<br>A: 38 (11.4), 18-63<br>G: 130 males, 111 females<br>O: 76% blue collar<br>H: Low back pain	1 week, 3 months	Moderate	Catastrophizing was positively related to days of absence (r = .23, *p* < .001) and negatively with work status (r = -.37, *p* < .001) in univariate analysis (correlations). Catastrophizing was only indirectly associated to days of absence and work status in multivariate analysis.
Carriere et al. 2015a<br>Canada<br>(North-America)	Prospective cohort study	N: 154<br>A: 36.4 (9.2), 20-60<br>G: 81 males, 73 females<br>O: Diverse<br>H: Whiplash associated disorders	1 year	Low	Participants who returned to work scored lower on catastrophizing than participants who did not RTW (t = 6.63, *p* < .001) in univariate analysis. Lower scores on pain catastrophizing (β = –.74, t(254) = –2.92, *p* = .004) were associated with successful RTW when controlling for RTW expectations. Pain catastrophizing also influences RTW expectations (*p* < .001). Expectations partially mediated the relation between pain catastrophizing and RTW status in multiple regression analysis.
Cowan et al. 2012<br>United States<br>(North-America)	Prospective cohort study	N: 66<br>A: 49.7 (11.3)<br>G: 17 males, 49 females<br>O: Desk-based, non-desk-based<br>H: Carpal tunnel release	2-4 months	Low	In bivariate analysis less catastrophic thinking (*p* = .005) was associated with earlier return to modified work. Pain catastrophizing was not significant for all subjects in predicting return to modified work, but was for desk-based subjects (R^2 = .15) in multivariate analysis.<br>In bivariate analysis less catastrophic thinking (*p* = .028) was associated with earlier return to full work. Pain catastrophizing was not a significant predictor of earlier return to full time work in multivariate analysis.
Dawson et al. 2011<br>Australia and New Zealand<br>(Oceania)	Cross-sectional study	No sick leave<br>N:1678<br>A: 45.2 (37.0-51.1), 18-65<br>G: 126 males, 1552 females<br>O: Nursing or midwives<br>H: Low back pain<br>Sick leave<br>N: 486<br>A: 43.9 (35.2-51.0), 18-65<br>G: 47 males, 439 females<br>O: Nursing or midwives<br>H: Low back pain	-	Moderate	Participants on sick leave had more pain catastrophizing (*p* < .001) than participants who were not on sick leave in bivariate analysis. Pain catastrophizing had no multivariable association with low back pain sick leave, possibly because it was correlated with passive coping and fear of movement.
De Vries et al.	Cross-	Staying at work	-	Low	Pain catastrophizing (higher in sick leave group) was associated with work status in univariate analysis (*p* = .001).

2012b<br>The Netherlands<br>(Europe)	sectional study	N: 119<br>A: 51, 20-60<br>G: 48 males, 71 females<br>O: -<br>H: Chronic nonspecific musculoskeletal pain<br>Sick leave and referred for rehabilitation<br>N: 122<br>A: 39, 20-60<br>G: 56 males, 66 females<br>O: -<br>H: Chronic nonspecific musculoskeletal pain			Participants who were on sick leave scored higher on pain catastrophizing than participants who stayed at work (B = -.07, OR: .93 (.88-.98), *p* = .005) in multivariate logistic regression analysis. However, catastrophizing was not one of the factors which best discriminated participants within the sick leave group and participants within the staying at work group in backwards stepwise logistic regression analysis.
Karels et al. 2010<br>The Netherlands<br>(Europe)	Prospective cohort study	N: 483<br>A: 41.5 (10.4)<br>G: 161 males, 322 females<br>O: Diverse<br>H: Arm, neck and shoulder complaints	3, 6 months	Moderate	High catastrophizing was associated with the occurrence of sickness absence in univariate analysis (OR: 2.8 (1.8-4.5), *p* < .05), but not in multivariate analysis because it was correlated with other factors as somatization.
Karoly et al. 2013<br>United States<br>(North-America)	Cross-sectional study	On disability<br>N: 434<br>A: 25-44:122, 45-64: 260, 65-80: 52<br>G: 221 males, 213 females<br>O: -<br>H: Chronic pain<br>Working<br>N: 859<br>A:25-44: 464, 45-64: 309, 65-80: 86<br>G: 481 males, 378 females<br>O: -<br>H: Chronic pain	-	Moderate	Participants who were not working scored higher on catastrophizing than participants who were working (*p* < .05) in univariate analysis (t-test). Catastrophizing (b = -.08, e: .92 (.90-.95), *p* < .001) was a negative predictor of continued employment after controlling for severity and demographic factors in multivariate logistic regression analysis.
Lindell et al. 2010<br>Sweden<br>(Europe)	Prospective cohort study	Rehabilitation group<br>N: 63<br>A: 42.2 (2.4), <60<br>G: 30 males, 33 females<br>O: 87% blue collar<br>H: non-acute non-specific spinal pain<br>Primary-care group<br>N: 62<br>A: 43.0 (2.6), <60<br>G: 27 males, 35 females<br>O: 87% blue collar<br>H: non-acute non-specific	6, 12, 18, 24 months	Low	Non-catastrophizing was a predictor of RTW at 18 months (*p* = .002) and at 24 months (*p* = .04), but not for RTW at 6 months and 12 months in univariate analysis.<br>Non-catastrophizing was only a predictor of RTW at 18 months (OR: 3.4 (1.3-9.1), *p* = .01) in multivariate logistic regression analysis, but not for RTW at 6 months, 12 months and 24 months. .

		spinal pain			
Mannion et al. 2009<br>Switzerland<br>(Europe)	Cross-sectional study	N: 670<br>A: 44.5 (10.2)<br>G: 362 males, 308 females<br>O: -<br>H: Low back pain	-	Moderate	Negative beliefs (thoughts about inevitable consequences) about low back pain were associated with more days off work (*p* = .0001) and reduced work-productivity (*p* = .0001) in univariate analysis with Spearman rank correlation coefficients. However, due to overlap between fear-avoidance beliefs and negative beliefs, negative beliefs was not a significant predictor of days of work absence in hierarchical multiple regression analysis.
Morris and Watson 2011<br>Jersey<br>(Europe)	Cross sectional study	Sick-listed<br>N: 62<br>A: 45.19 (10.41), >18<br>G: 40 males, 22 females<br>O: Private sector: 45, public: sector: 17<br>H: Low back pain<br>Non-sick-listed<br>N: 57<br>A: 46.04 (11.98), >18<br>G: 29 males, 28 females<br>O: Private sector: 45, public sector: 11<br>H: Low back pain	-	Low	There was no significant difference between the non-sick listed and sick-listed participants beliefs about the inevitable consequences of low back pain in univariate analysis.
Sarda et al. 2009<br>Australia<br>(Oceania)<br>Brazil<br>(South-America)	Cross-sectional study	Australian<br>N: 207<br>A: 44, 18-65<br>G: -<br>O: -<br>H: Chronic pain<br>Brazil<br>N: 222<br>A: 45, 18-65<br>G: -<br>O: -<br>H: Chronic pain	-	Low	Catastrophizing was not associated with unemployment in the Australian and Brazilian sample in multivariate logistic regression analysis.
Spector et al. 2012<br>United States<br>(North-America)	Prospective cohort study	N: 670<br>A: 44.9 (9.6), >18<br>G: 255 males, 415 females<br>O: Diverse<br>H: Carpal tunnel syndrome	2 years	Low	A high score on catastrophizing was associated with higher odds of long-term disability (missing work days and retrieving compensation) in univariate analysis ($p < .01$), but was not significant in the final multi-domain model in multivariate analysis.
Turner et al. 2008<br>United States<br>(North-America)	Prospective cohort study	N: 1885<br>A: 39.4 (11.2)<br>G: 1282 males, 603 females<br>O: Diverse<br>H: Back injury	1 year	Low	Catastrophizing was associated with 1-year work disability (wage replacement compensation for total disability 12 months after claim submission) in univariate analysis ($p < .001$). In a multi-domain model none of the psychological factors, including catastrophizing, contributed independently to the prediction of 1-year work disability. This was because the score on the Roland-Morris disability questionnaire was substantially correlated with all the psychological factors.
Wijnhoven et al. 2007<br>The Netherlands<br>(Europe)	Cross-sectional study	N: 2517<br>A: 25 – 64<br>G: 1070 males, 1447 females<br>O: -	-	Moderate	For men with paid work high pain catastrophizing was positively associated with work leave in the past 12 months (PR: 1.56 (1.11-2.19), $p < .05$). For women with paid work medium catastrophizing (PR: 1.80 (1.14-2.85), $p < .05$) and high catastrophizing (PR: 1.69 (1.06-2.69), $p < .05$) were positively associated with work leave in the past 12 months in log-binomial regression analysis.

		H: Musculoskeletal pain			

* RTW: Return to work
