# Supplementary material for: Person-related factors associated with work participation in employees with health problems: a systematic review
Source: Int Arch Occup Environ Health. 2018 Apr 26;91(5):497–512. doi: 10.1007/s00420-018-1308-5 (PMC6002456; doi:10.1007/s00420-018-1308-5)
Supplement: Supplementary file 3 — Supplementary material 3 (PDF 362 KB) [file 420_2018_1308_MOESM3_ESM.pdf]

Person-related factors associated with work participation in employees with health problems: a systematic review

M. de Wit, H. Wind, C. T. J. Hulshof, M. H. W. Frings-Dresen

Department Coronel Institute of Occupational Health, Academic Medical Center, University of Amsterdam, Amsterdam Public Health research institute, Amsterdam, the Netherlands

Correspondence to: Mariska de Wit, Department Coronel Institute of Occupational Health, Academic Medical Center, University of Amsterdam, Amsterdam Public Health research institute, PO Box 22700, NL-1100 DE Amsterdam, The Netherlands; m.e.dewit@amc.uva.nl; +31(0)20-5665341

Journal: International Archives of Occupational and Environmental Health

Online Resource 3

Online Resource 3

Scores on the criteria of the Joanna Briggs Institute quality assessment tools for the included articles

Study	Study Design	Q1	Q2	Q3	Q4	Q5	Q6	Q7	Q8	Q9	Q10	Q11	Q12	Q13	Quality score /total	Risk of bias
Adams et al. 2017	PCS	1	1	1	1	1	1	1	0	1	X	1	X	X	10/11	Low
Åhrberg et al. 2010	QS	1	1	1	1	1	0	0	1	1	1	X	X	X	8/10	Moderate
Arwert et al. 2017	CSS	1	1	1	1	1	1	1	1	X	X	X	X	X	8/8	Low
Audhoe et al. 2012	PCS	1	1	0	1	1	1	1	1	0	1	1	X	X	9/11	Low
Awang et al. 2016	CSS	0	1	0	1	1	1	1	1	X	X	X	X	X	6/8	Moderate
Becker et al. 2007	QS	0	1	1	1	1	0	1	0	1	1	X	X	X	7/10	Moderate
Besen et al. 2015	PCS	1	1	1	1	1	1	0	1	0	0	1	X	X	8/11	Moderate
Boot et al. 2008	CSS	1	1	1	1	1	1	1	1	X	X	X	X	X	8/8	Low
Boot et al. 2014	PCS	1	1	1	1	1	1	1	1	0	0	1	X	X	9/11	Low
Boot et al. 2011	CSS	1	1	0	1	1	1	0	1	X	X	X	X	X	6/8	Moderate
Boyle et al. 2014	CSS	1	1	1	1	1	1	1	1	X	X	X	X	X	8/8	Low
Braathen et al. 2007	non-RCT	1	1	0	1	0	0	1	0	1	X	X	X	X	5/9	Moderate
Brouwer et al. 2015	PCS	1	1	1	1	1	1	1	1	1	1	1	X	X	11/11	Low
Brouwer et al. 2009	PCS	1	1	1	1	1	1	1	1	1	X	1	X	X	11/11	Low
Brouwer et al. 2010	PCS	1	1	1	1	1	1	1	1	0	1	1	X	X	10/11	Low
Busch et al. 2007	PCS	1	1	1	1	1	0	1	1	1	X	1	X	X	10/11	Low
Carriere et al. 2015a	PCS	1	1	1	1	1	1	1	1	0	0	1	X	X	9/11	Low
Carriere et al. 2015b	PCS	1	1	1	1	1	1	1	1	0	1	1	X	X	10/11	Low
Carstens et al. 2014	PCS	1	1	1	0	0	0	1	1	0	0	1	X	X	6/11	Moderate
Chen et al. 2012	CCS	1	1	1	1	1	1	0	1	X	X	X	X	X	7/8	Low
Coggon et al. 2013	CSS	1	1	1	0	1	1	0	1	X	X	X	X	X	6/8	Moderate
Cowan et al. 2012	PCS	1	1	1	1	1	1	0	1	1	1	1	X	X	10/11	Low

Study	Study Design	Q1	Q2	Q3	Q4	Q5	Q6	Q7	Q8	Q9	Q10	Q11	Q12	Q13	Quality score /total	Risk of bias
D’Amato and Zijlstra 2010	PCS	0	1	1	1	1	0	1	1	0	0	1	X	X	7/11	Moderate
Dawson et al. 2011	CSS	1	1	1	0	1	1	0	1	X	X	X	X	X	6/8	Moderate
De Vries et al. 2011	QS	1	1	1	1	1	1	1	1	1	1	X	X	X	10/10	Low
De Vries et al. 2012b	CSS	1	1	1	1	1	1	0	1	X	X	X	X	X	7/8	Low
Dekkers-Sánchez et al. 2010	QS	1	1	1	1	1	0	0	1	1	1	X	X	X	8/10	Moderate
Dionne et al. 2013	QS	0	1	1	1	1	1	0	1	1	1	X	X	X	8/10	Moderate
Dionne et al. 2007	PCS	1	0	1	1	1	0	1	1	1	X	1	X	X	9/11	Low
Du Bois et al. 2009	PCS	1	0	1	1	1	1	0	1	1	X	1	X	X	9/11	Low
Dunn et al. 2010	QS	1	1	1	1	1	0	0	1	1	1	X	X	X	8/10	Moderate
Dyster-Aas et al. 2007	CSS	1	1	1	1	1	1	0	1	X	X	X	X	X	7/8	Low
Ekberg et al. 2015	PCS	1	1	1	1	1	1	1	1	0	1	1	X	X	10/11	Low
Elfving et al. 2009	PCS	1	1	1	1	1	0	0	1	1	X	1	X	X	9/11	Low
Ellingsen and Aas 2009	QS	1	1	1	1	1	0	0	1	1	1	X	X	X	8/10	Moderate
Gross and Battié 2010	PCS	1	1	1	1	1	1	0	1	0	0	1	X	X	8/11	Moderate
Grøvle et al. 2013	PCS	1	1	1	1	1	1	1	1	1	X	1	X	X	11/11	Low
Grytten et al. 2017	PCS	1	1	1	1	1	1	0	1	1	X	1	X	X	10/11	Low
Hansen et al. 2009	PCS	1	1	0	1	1	0	0	1	1	X	1	X	X	8/11	Moderate
Hartke et al. 2011	QS	1	1	1	1	1	0	0	1	0	1	X	X	X	7/10	Moderate
He et al. 2010	RCS	1	1	0	1	1	1	1	1	1	X	1	X	X	9/11	Low
Healey et al. 2011	CSS	1	1	1	1	1	1	1	1	X	X	X	X	X	8/8	Low
Heymans et al. 2009	PCS	0	1	1	1	1	1	1	1	1	X	1	X	X	11/11	Low
Heymans et al. 2007	RCS	1	1	1	1	1	0	1	1	1	X	1	X	X	10/11	Low
Hou et al. 2012	PCS	1	1	1	1	1	1	1	1	0	0	1	X	X	9/11	Low
Hou et al. 2008	PCS	1	1	0	1	1	1	1	1	1	X	1	X	X	10/11	Low
Huijs et al. 2012	PCS	1	1	1	1	1	1	1	1	0	0	1	X	X	9/11	Low
Huijs et al. 2017	PCS	1	1	1	1	1	0	1	1	0	0	1	X	X	8/11	Moderate

Study	Study Design	Q1	Q2	Q3	Q4	Q5	Q6	Q7	Q8	Q9	Q10	Q11	Q12	Q13	Quality score /total	Risk of bias
Hystad and Bye 2012	PCS	1	1	1	1	1	0	1	1	1	X	1	X	X	10/11	Low
Iakova et al. 2012	PCS	1	1	1	1	1	0	1	1	0	0	1	X	X	8/11	Moderate
Jensen et al. 2013	PCS	1	1	0	1	1	1	1	1	1	X	1	X	X	10/11	Low
Johansson et al. 2010	PCS	1	1	1	1	1	0	1	1	1	X	1	X	X	10/11	Low
Karels et al. 2010	PCS	1	1	1	1	1	0	1	1	0	0	1	X	X	8/11	Moderate
Karoly et al. 2013	CSS	1	1	1	0	1	1	0	1	X	X	X	X	X	6/8	Moderate
Kovacs et al. 2007	PCS	1	1	1	1	1	0	1	1	1	X	1	X	X	10/11	Low
Krause et al. 2013	CSS	1	1	1	1	0	0	0	1	X	X	X	X	X	5/8	Moderate
Kuijer et al. 2016	RCS	1	1	1	1	1	1	1	1	0	0	1	X	X	9/11	Low
Lagerveld et al. 2016	PCS	0	0	1	1	1	1	1	1	0	0	1	X	X	7/11	Moderate
Lindell et al. 2010	PCS	1	1	1	1	1	1	0	1	1	X	1	X	X	10/11	Low
Luk et al. 2010	PCS	1	1	1	1	1	1	0	1	1	X	1	X	X	9/11	Low
Lundqvist and Sumuelsson 2012	QS	1	1	1	1	1	0	0	1	1	1	X	X	X	8/10	Moderate
Lydell et al. 2011	PCS	1	0	0	0	0	1	1	1	1	X	1	X	X	7/11	Moderate
Magnussen et al. 2007a	QS	1	1	1	1	1	0	1	1	0	1	X	X	X	8/10	Moderate
Magnussen et al. 2007b	RCT	1	1	1	0	0	0	1	1	0	1	1	1	0	8/13	Moderate
Mangels et al. 2011	PCS	1	1	1	0	0	1	0	1	1	X	1	X	X	8/11	Moderate
Mannion et al. 2009	CSS	0	1	1	0	1	1	1	1	X	X	X	X	X	6/8	Moderate
Martins 2015	CSS	1	1	1	1	0	0	0	1	X	X	X	X	X	5/8	Moderate
Morris and Watson 2011	CSS	1	1	1	1	1	1	1	1	X	X	X	X	X	8/8	Low
Morrison et al. 2016	PCS	1	0	0	1	1	0	1	1	0	0	1	X	X	6/11	Moderate
Murgatroyd et al. 2016	PCS	1	1	1	1	1	0	1	1	0	0	1	X	X	8/11	Moderate
Murphy et al. 2011	PCS	1	1	0	0	0	0	1	1	1	X	1	X	X	7/11	Moderate
Nielsen et al. 2012	PCS	1	0	0	1	1	1	1	1	1	X	1	X	X	9/11	Low
Nieuwenhuijsen et al. 2013	RCS	0	1	1	1	1	1	1	0	0	0	1	X	X	7/11	Moderate
Norlund et al. 2011	PCS	1	1	1	1	1	1	1	1	1	X	1	X	X	11/11	Low

Study	Study Design	Q1	Q2	Q3	Q4	Q5	Q6	Q7	Q8	Q9	Q10	Q11	Q12	Q13	Quality score /total	Risk of bias
Opsahl et al. 2016	PCS	1	1	1	1	1	1	0	1	1	X	1	X	X	10/11	Low
O'Sullivan et al. 2012	CSS	1	1	1	1	1	1	0	1	X	X	X	X	X	7/8	Low
Øyeflaten et al. 2008	PCS	1	1	1	1	1	1	1	1	0	0	1	X	X	9/11	Low
Øyeflaten et al. 2016	PCS	0	1	1	0	0	1	1	1	1	X	1	X	X	8/11	Moderate
Poulain et al. 2010	PCS	1	1	1	1	1	1	1	1	0	1	1	X	X	10/11	Low
Puolakka et al. 2008	PCS	1	1	1	1	1	1	0	1	1	X	1	X	X	10/11	Low
Ramel et al. 2013	PCS	1	1	1	1	1	1	0	1	1	X	1	X	X	10/11	Low
Reme et al. 2009	PCS	1	1	1	1	1	1	1	1	1	0	1	X	X	10/11	Low
Richard et al. 2011	PCS	1	1	1	1	1	1	1	1	0	0	1	X	X	9/11	Low
Richter et al. 2011	PCS	1	1	0	1	1	1	1	1	1	X	1	X	X	10/11	Low
Roesler et al. 2013	PCS	1	1	1	1	1	1	1	1	1	X	1	X	X	11/11	Low
Rönnberg et al. 2007	PCS	1	1	1	0	0	1	1	1	0	0	1	X	X	7/11	Moderate
Sampere et al. 2012	PCS	1	1	1	1	1	1	1	1	0	0	1	X	X	9/11	Low
Saperstein et al. 2011	CSS	1	1	1	1	0	0	1	1	X	X	X	X	X	6/8	Moderate
Sarda et al. 2009	CSS	1	1	1	1	1	1	0	1	X	X	X	X	X	7/8	Low
Selander et al. 2007	PCS	1	1	1	1	1	0	1	1	1	X	1	X	X	10/11	Low
Shaw et al. 2011	PCS	1	1	0	1	1	0	1	1	0	0	1	X	X	7/11	Moderate
Sivertsen et al. 2013	CSS	1	1	0	1	1	1	1	1	X	X	X	X	X	7/8	Low
Sluiter and Frings-Dresen 2008	CSS	1	1	1	1	1	1	1	1	X	X	X	X	X	8/8	Low
Spector et al. 2012	PCS	1	1	1	1	1	0	1	1	1	1	1	X	X	10/11	Low
Steenstra et al. 2010	CSS	1	1	1	1	1	1	1	1	X	X	X	X	X	8/8	Low
Strauser et al. 2010	CSS	1	1	1	1	1	1	0	1	X	X	X	X	X	7/8	Low
Strober and Arnett 2016	CSS	1	1	1	1	0	0	1	1	X	X	X	X	X	6/8	Moderate
Stulemeijer et al. 2008	PCS	1	1	1	1	1	1	1	1	0	0	1	X	X	9/11	Low
Tamminga et al. 2012	QS	1	1	1	1	1	1	0	1	1	1	X	X	X	9/10	Low
Torres et al. 2009	PCS	1	1	1	1	1	0	1	1	1	X	1	X	X	10/11	Low

Study	Study Design	Q1	Q2	Q3	Q4	Q5	Q6	Q7	Q8	Q9	Q10	Q11	Q12	Q13	Quality score /total	Risk of bias
Truchon et al. 2010	PCS	1	1	1	1	1	1	0	1	0	0	1	X	X	8/11	Moderate
Truchon et al. 2012	PCS	1	1	0	0	0	1	0	1	0	0	1	X	X	5/11	High
Turner et al. 2008	PCS	1	0	0	1	1	1	1	1	1	X	1	X	X	9/11	Low
Van Velzen et al. 2011	QS	1	1	1	1	1	0	0	1	0	1	X	X	X	7/10	Moderate
Vlasveld et al. 2013	CSS	1	1	1	1	1	1	1	1	X	X	X	X	X	8/8	Low
Volker et al. 2015	PCS	1	1	1	1	1	1	1	1	1	X	1	X	X	11/11	Low
Vuistiner et al. 2015	PCS	1	0	1	1	1	1	0	1	1	X	1	X	X	9/11	Low
Waghorn et al. 2007	PCS	1	1	0	1	1	0	0	1	1	X	1	X	X	8/11	Moderate
Wåhlin et al. 2012	PCS	1	1	1	1	1	1	1	1	1	X	1	X	X	11/11	Low
Wan Kasim et al. 2014	CSS	1	1	0	1	1	1	1	1	X	X	X	X	X	7/8	Low
Waynor et al. 2016	PCS	1	1	1	0	0	1	0	1	0	1	1	X	X	7/11	Moderate
Wijnhoven et al. 2007	CSS	1	1	1	0	1	1	0	1	X	X	X	X	X	6/8	Moderate
Wilbanks and Ivankova 2015	QS	1	1	1	1	1	1	1	1	1	1	X	X	X	10/10	Low

* RCT: Randomized Controlled Trial, PCS: Prospective Cohort Study, RCS: Retrospective Cohort Study, CSS: Cross-sectional Study, QS: Qualitative Study

X, item not applicable; 1, description in study meets criterion; 0, description in text does not meet criterion
